# Efficacy and safety of corticosteroids in cardiac arrest: a systematic review, meta-analysis and trial sequential analysis of randomized control trials

**DOI:** 10.1186/s13054-022-04297-2

**Published:** 2023-01-11

**Authors:** Jeremy Penn, Will Douglas, Jeffrey Curran, Dipayan Chaudhuri, Joanna C. Dionne, Shannon M. Fernando, David Granton, Rebecca Mathew, Bram Rochwerg

**Affiliations:** 1 Department of Medicine, Division of Critical Care, Juravinski Hospital, McMaster University, 711 Concession St, Hamilton, ON L8V 1C1 Canada; 2 Department of Critical Care Medicine, Queen’s University, Kingston, Canada; 3 Division of Critical Care, Department of Medicine, University of Ottawa, Ottawa, ON Canada; 4 Department of Critical Care, Lakeridge Health Corporation, Oshawa, ON Canada; 5 Department of Medicine, University of Toronto, Toronto, ON Canada; 6 Division of Cardiology, Department of Medicine, University of Ottawa Heart Institute, Ottawa, ON Canada

**Keywords:** Cardiac arrest, Corticosteroids, Randomized control trial, Mortality, Survival with good functional outcome

## Abstract

**Background:**

Post-cardiac arrest, outcomes for most patients are poor, regardless of setting. Many patients who do achieve spontaneous return of circulation require vasopressor therapy to maintain organ perfusion. There is some evidence to support the use of corticosteroids in cardiac arrest.

**Research question:**

Assess the efficacy and safety of corticosteroids in patients following in- and out-of-hospital cardiac arrest.

**Study design and methods:**

We searched databases CINAHL, EMBASE, LILACS, MEDLINE, Web of Science, CENTRAL, ClinicalTrails.gov, and ICTRP. We included randomized controlled trials (RCTs) that examined the efficacy and safety of corticosteroids, as compared to placebo or usual care in patients post-cardiac arrest. We pooled estimates of effect size using random effects meta-analysis and report relative risk (RR) with 95% confidence intervals (CIs). We assessed risk of bias (ROB) for the included trials using the modified Cochrane ROB tool and rated the certainty of evidence using Grading of Recommendations Assessment, Development and Evaluation methodology.

**Results:**

We included 8 RCTs (*n* = 2213 patients). Corticosteroids administered post-cardiac arrest had an uncertain effect on mortality measured at the longest point of follow-up (RR 0.96, 95% CI 0.90–1.02, very low certainty, required information size not met using trial sequential analysis). Corticosteroids probably increase return of spontaneous circulation (ROSC) (RR 1.32, 95% CI 1.18–1.47, moderate certainty) and may increase the likelihood of survival with good functional outcome (RR 1.49, 95% CI 0.87–2.54, low certainty). Corticosteroids may decrease the risk of ventilator associated pneumonia (RR 0.76, 95% CI 0.46–1.09, low certainty), may increase renal failure (RR 1.29, 95% CI 0.84–1.99, low certainty), and have an uncertain effect on bleeding (RR 2.04, 95% CI 0.53–7.84, very low certainty) and peritonitis (RR 10.54, 95% CI 2.99–37.19, very low certainty).

**Conclusions:**

In patients during or after cardiac arrest, corticosteroids have an uncertain effect on mortality but probably increase ROSC and may increase the likelihood of survival with good functional outcome at hospital discharge. Corticosteroids may decrease ventilator associated pneumonia, may increase renal failure, and have an uncertain effect on bleeding and peritonitis. However, the pooled evidence examining these outcomes was sparse and imprecision contributed to low or very low certainty of evidence.

**Supplementary Information:**

The online version contains supplementary material available at 10.1186/s13054-022-04297-2.

## Introduction

Outcomes following cardiac arrest, either in-hospital or out-of-hospital, are poor [1, 2]. Cardiac arrest is associated with high mortality, and even among survivors, hypoxic-ischemic brain injury and resultant functional disability are common [3, 4]. In those who achieve spontaneous return of circulation (ROSC), hemodynamic instability occurs in at least 40% of patients in the peri- and post-resuscitative period, and patients often require vasopressor therapy to maintain adequate mean arterial pressures and maintain organ perfusion [5]. The etiology of post-arrest hypotension is multifactorial, including massive inflammatory response secondary to cardiac arrest, prolonged tissue ischemia, myocardial stunning, and relative adrenal insufficiency [6].

There is some evidence supporting the administration of corticosteroids during acute resuscitation in cardiac arrest. Although the mechanism of action for corticosteroids in cardiac arrest remains uncertain, their ability to downregulate systemic inflammation may reduce time to shock resolution and improve survival. There are a number of small randomized controlled trials (RCTs) addressing this question; however, clinical uncertainty persists as to whether patients post-cardiac arrest should receive corticosteroids, and clinical practice remains varied [7–10]. The objective of this systematic review and meta-analysis is to summarize RCTs evaluating the efficacy and safety of corticosteroids in patients during and immediately following cardiac arrest.

## Methods

We registered the protocol for this systematic review on PROSPERO December 12, 2020 (CRD42020221818).

### Data sources and searches

We searched CINAHL, EMBASE, LILACS, MEDLINE, Web of Science, CENTRAL, ClinicalTrails.gov, and ICTRP for RCTs published from database inception until June 1, 2022. We developed the search strategy in consultation with an experienced health science librarian. We included the keywords “cardiac arrest” or “cardiopulmonary arrest” or “circulation arrest” or “circulatory arrest” and a number of corticosteroids including but not limited to “prednisolone” or “prednisolone” or “methylprednisolone” or “hydrocortisone” or “aldosterone” (see Additional file 1: Appendix 1–6 for full search strategy).

### Study selection

We screened all citations independently and in duplicate. Reviewers (JP, WD, JC) initially screened titles and abstracts, and any citation identified as potentially relevant by either reviewer was advanced to full text review. Disagreements were resolved through discussion or fourth-person adjudication (BR). We captured reasons for full text exclusion.

We included RCTs comparing the use of intravenous corticosteroids with placebo or standard care in adult patients (> 18 years of age) during or immediately following cardiac arrest (any initial rhythm or etiology), regardless of whether the arrest occurred in- or out-of-hospital. We examined the following outcomes: mortality (at the longest time of follow-up), ROSC, survival with good functional outcome, ventilator associated pneumonia, bleeding, peritonitis, and acute renal failure (all as defined by study authors). We did not employ any exclusion criteria based on language of publication.

### Data abstraction and quality assessment

Three reviewers performed data extraction independently and in duplicate using predefined data abstraction forms (JP, WD, JC). A fourth reviewer resolved disagreements (BR). We abstracted the following data: study characteristics, demographic data, intervention and control details, and outcome data [11].

We assessed individual study risk of bias (ROB) independently and in duplicate using the modified Cochrane ROB tool. The tool classifies ROB as "low," "probably low," "probably high," and "high" for the following criteria: sequence generation, allocation concealment, blinding, selective outcome reporting, and other bias [12]. We rated overall study ROB as the highest risk attributed to any of the assessed criteria. We assessed overall certainty of evidence for each outcome using the Grading of Recommendations Assessment, Development, and Evaluation (GRADE) framework [13]. The GRADE system provides a framework for the assessment of certainty of evidence for each individual outcome. The GRADE approach specifies four levels of certainty: "High," "Moderate," "Low," and "Very Low." Disagreements with respect to ROB and GRADE assessments were resolved by discussion [13]. As recommended by GRADE guidance, we applied informative narrative statements (“probably,” “possibly,” “may”) to communicate our confidence in the effect estimates [14]. We performed this meta-analysis in accordance with the latest PRISMA guidance (see Additional file 1: Appendix 11 for completed checklist) [15].

### Data analysis

We performed all analyses using RevMan 5.4.1 (Cochrane Collaboration, Oxford) software [16]. We used the DerSimonian-Laird random effects model with inverse-variance weighting to generate pooled treatment effects across RCTs. We assessed statistical heterogeneity between trials using a combination of the *χ*^2^ test for homogeneity, the *I*^2^ test, and visual inspection of the forest plots. We presented results of dichotomous outcomes using relative risk (RR) with a 95% confidence interval (CI). We conducted trial sequential analysis (TSA) using a random effects model for the outcome of mortality (see Additional file 1: Appendix 10). For the TSA, we used a statistical significance level of 5%, a power of 80%, and a relative risk reduction of 15%. We used a model variance-based heterogeneity corrected [17]. We performed TSA using trial sequential analysis v.0.9.5.10 beta (Copenhagen Trial Unit, Centre for Clinical Intervention Research, Rigshospitalet, Copenhagen, Denmark, www.ctu.dk/tsa).

We identified five a priori subgroups of interest: high ROB versus low ROB studies, corticosteroid type (hydrocortisone vs. methylprednisolone vs. dexamethasone), initiation of corticosteroids after cardiac arrest (following ROSC) versus during cardiac arrest (during CPR), corticosteroid dose (high vs. low based on whether the dose was above or below the mean dose used across included studies), and in-hospital cardiac arrest (IHCA) versus out-of-hospital cardiac arrest (OHCA).

## Results

### Trial characteristics

**Fig. 1:**
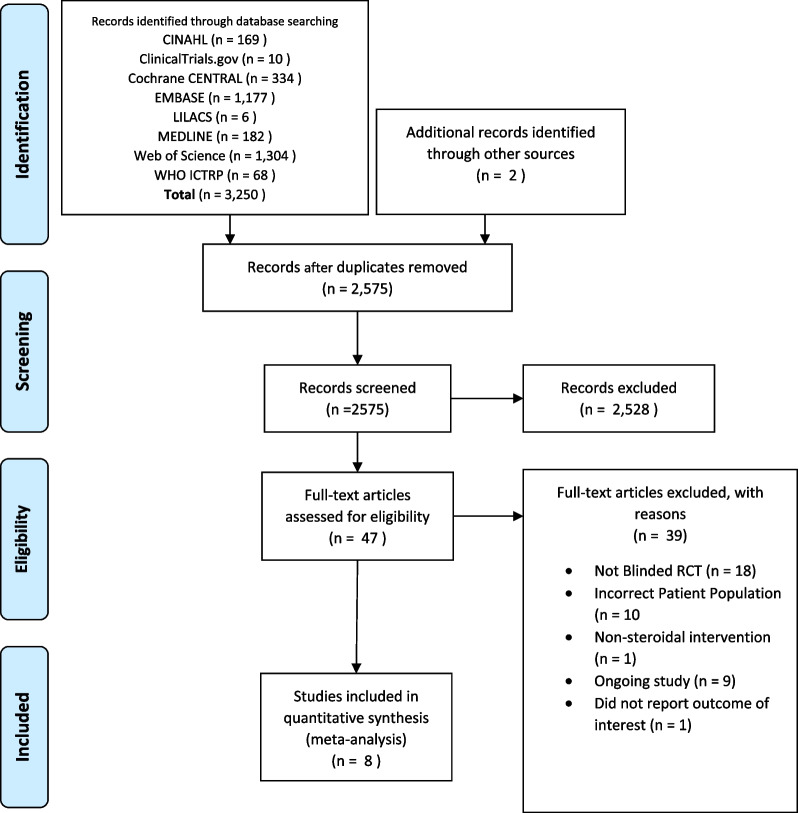
PRISMA flow—study inclusion

Of the initial 3250 citations, we reviewed 47 full texts and included 8 RCTs examining 2213 patients which met eligibility criteria [7–10, 18–21]. We excluded 1 abstract as it did not report any of the outcomes of interest [22] (Fig. 1). Trials randomized between 50 and 814 patients; 4 trials were conducted at a single center (one of which collected patients from 13 mobile ICUs connected to a single hospital) [8, 10, 20, 21] while 3 others were multi-site studies ranging from 3 to 10 centers. One trial did not report the number of centers [17]. Six of the eight trials were blinded [7–9, 18–20]; two trials were not blinded [10, 21].

Trials were performed in the USA (*n* = 3), Greece (*n* = 2), Iran (*n* = 2), and Denmark (*n* = 1); 6 trials examined only in-hospital cardiac arrest [7, 8, 10, 18, 20, 21] while 1 trial included mostly out-of-hospital cardiac arrests (76%) [9]. Another trial did not report whether arrests were in-hospital or out-of-hospital [19]. A total of 6 trials used methylprednisone [7, 8, 10, 18, 19, 21], 1 trial used dexamethasone [20], and 1 trial used hydrocortisone [10]. Additionally, steroid dose varied among the trials with a median dose of 40 mg as a methylprednisone equivalent (interquartile range = 110). Only 1 trial calculated corticosteroid dose based on actual body weight as 30 mg/kg of methylprednisone with a maximum dose of 3 g [19]. Two trials administered vasopressin at 20 units/CPR cycle as part of the intervention in addition to corticosteroids [7, 8]. One trial did not report the amount of vasopressin administered [21]. Further trial characteristics are presented in Table 1.

**Table 1:** Summary of baseline characteristics of randomized control trials

First author, year	Trial registration	Sample size (study group: control)	Country leading study	Number of centers	Enrolment period	Funding	Location of cardiac arrest	Time of intervention administration	Study group	Control group
Paris [20]	NR	81 (37:46)	USA	Single—(13 Mobile Intensive Care Units)	November, 1984	Organon Pharmaceuticals	100% out-of-hospital	During CPR	Patients were given 100 mg of dexamethasone in 10 cc of diluent during CPR	Patients were given an identical study group matched volume of placebo during CPR
Metz [19]	NR	814 (418:396)	USA	NR	NR	Grant from the Upjohn Company (Former Pharmaceutical Company)	NR	After CPR	Patients received investigational therapy < 6 h post-arrest and 30 mg/kg methylprednisolone sodium succinate (3 g maximum)	Patients received investigational therapy 6–12 h post-arrest and 30 mg/kg of an identical study group matching placebo (3 g maximum)
Mentzelopoulos [8]	NCT00411879	100 (48:52)	Greece	1	07/2006–03/2007	Thorax Research Foundation, Athens, Greece, and the Greek Society of Intensive Care Medicine	100% In-hospital	During and after CPR	Patients were given vasopressin (20 IU/CPR cycle) plus epinephrine (1 mg per CPR cycle) for the first 5 cycles post-randomization. During the first cycle of CPR, patients received methylprednisolone (40 mg). Shock after resuscitation was treated with stress-dose hydrocortisone (300 mg daily for 7 days maximum and gradual taper)	Patients were given isotonic sodium chloride solution placebo solution with epinephrine (1 mg per CPR cycle) for the first 5 CPR cycles post-randomization On the first CPR cycle, patients received saline placebo. Shock was treated with stress-dose hydrocortisone-sodium succinate (300 mg daily for 7 days maxi- mum, with gradual taper)
Mentzelopoulos [7]	NCT00729794	268 (130/− 138)	Greece	3	09/2008–10/2010	Greek Society of Intensive Care Medicine and the Project “Synergasia” (ie, Cooperation) of the Greek Ministry of Education	100% In-hospital	During and after resuscitation	Patients were given vasopressin (20 IU/CPR cycle) plus epinephrine (1 mg per CPR cycle; cycle duration approximately 3 min) for the first 5 CPR cycles after randomization, followed by additional epinephrine if needed. During the first cycle of CPR, patients received d methylprednisolone (40 mg). Shock after resuscitation was treated with stress-dose hydrocortisone (300 mg daily for 7 days maximum and gradual taper)	Patients were given saline placebo plus epinephrine (1 mg per CPR cycle; cycle duration approximately 3 min) for the first 5 CPR cycles after randomization, followed by additional epinephrine if needed. During the first CPR cycle after randomization, patients received saline placebo. Shock after resuscitation was treated with saline placebo
Donnino [9]	NCT00676585	50 (25:25)	USA	3	01/2008–3/2014	Grant from the American Heart Association	24% in-hospital: 76% out-of hospital	During and After CPR	Patients were given 100 mg hydrocortisone intravenously every 8 h up to 7 days or 24 h after shock reversal	Patients were given identical study group matched volumes of placebo intravenously every 8 h up to 7 days or 24 h after shock reversal
Bolvardi [10]	NR	50 (25:25)	Iran	1	2015	NR	100% in-hospital	During CPR (first cycle)	Patients were given epinephrine (1mp per CPR cycle) and 150 mg intravenous methylprednisone during the first cycle of CPR or after the second administration of epinephrine	Patients were given epinephrine (1mp per CPR cycle) and identical study group matched volumes of saline as placebo were administered during the first cycle of CPR or after the second administration of epinephrine
Andersen [1]	NCT03640949	501 (237:264)	Denmark	10	October 15, 2018, to January 21, 2021	Aarhus University Research Foundation; the Department of Clinical Medicine, Aarhus University; the Central Denmark Region; and the Independent Research Fund Denmark	100% in-hospital	NR	Patients were given (40 mg) of methylprednisolone and 20 IU of vasopressin after the first dose of epinephrine. Additional doses of vasopressin (20 IU) were administered after each epinephrine dose for a maximum of 4 doses (80 IU)	Patients were given 9 mg/mL of sodium chloride from identical ampoules after each epinephrine dose for a maximum of 4 doses (80 IU)
Rafiei [21]	IRCT20130812014333N127	347 (171:176)	Iran	1	May 15, 2019 – August 16, 2019	Kermanshah University of Medical Sciences	100% in-hospital	During and after CPR	Patients received epinephrine at a dose of 1 mg per CPR cycle and 125 mg methylprednisone during the first cycle of resuscitation or during the second injection of epinephrine	Patients received epinephrine at a dose of 1 mg per CPR cycle and a placebo control during the first cycle of resuscitation or during the second injection of epinephrine

### Risk of bias

Four trials were low ROB [7, 8, 18, 21], and 4 trials were high ROB [9, 10, 19, 20]. Of the high ROB trials, 1 did not specify their blinding methods [10]. Only 1 trial did not report any blinding of its outcome assessors [10]. All high ROB trials did not describe their allocation concealment [9, 10, 19, 20]. See Additional file 1: Appendix 7 for complete ROB assessment.

### Outcomes

**Fig. 2:**
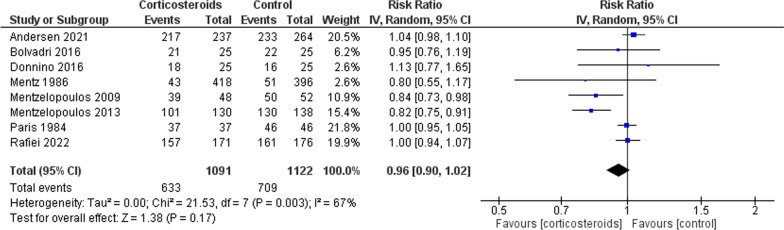
Comparing corticosteroids and placebo for the outcome of mortality closest to 28 days; results are shown by using the random-effects model with relative risk and 95% confidence intervals (CI)

**Fig. 3:**
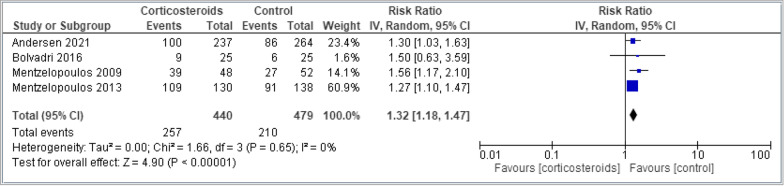
Comparing corticosteroids and placebo for the outcome of return of spontaneous circulation; results are shown by using the random-effects model with relative risk and 95% confidence intervals (CI)

**Fig. 4:**
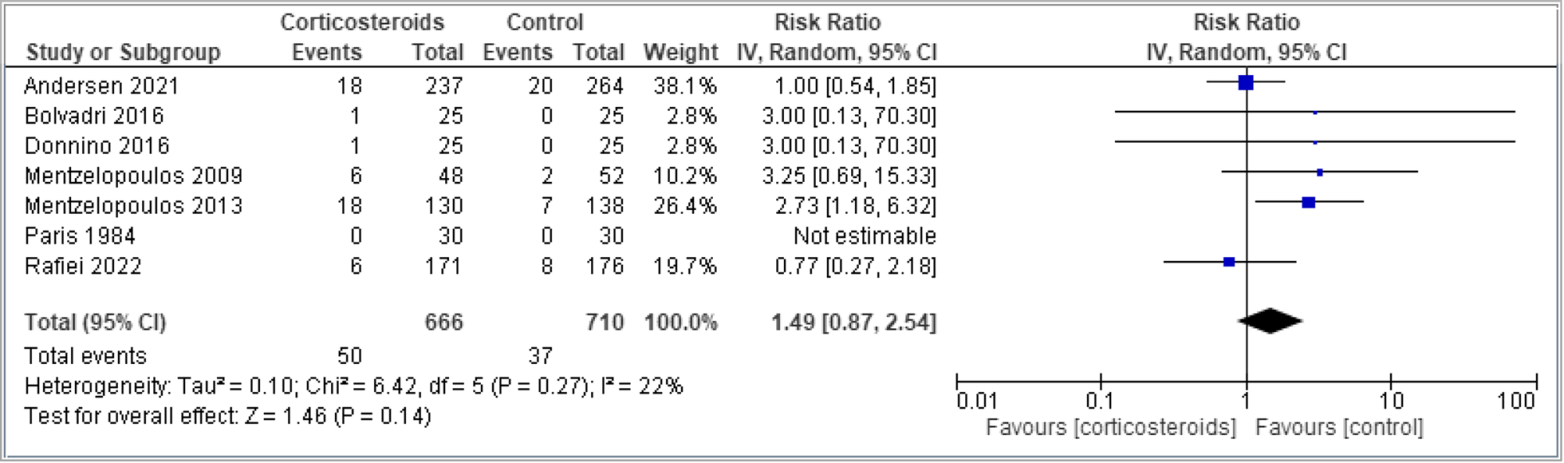
Comparing corticosteroids and placebo for the outcome of survival with good functional outcome; results are shown by using the random-effects model with relative risk and 95% confidence intervals (CI)

Table 2 shows the summary of findings for all outcomes including the certainty of evidence and reasons for rating down the evidence. Corticosteroids administered in the setting of cardiac arrest have an uncertain effect on mortality measured at the longest point of follow-up (8 trials, 2213 patients, RR 0.96, 95% CI 0.90–1.02, I2 67%, very low certainty) (Fig. 2). The TSA showed the required information size was not met. Corticosteroids probably increase ROSC (4 trials, 919 patients, RR 1.32, 95% CI 1.18–1.47, I2 0%, moderate certainty) (Fig. 3) and may increase the likelihood of survival with good functional outcome (6 trials, 1,029 patients, RR 1.40, 95% CI 0.87–2.54, I2 22%, low certainty) (Fig. 4). Survival with good functional outcome at hospital discharge was determined using the Glasgow Pittsburgh Cerebral Performance Category (CPC) for all trials [7, 8, 10, 18, 21]. Four trials defined survival with good functional outcome as a CPC score of 1 (conscious with normal function or only slight disability) or 2 (conscious with moderate disability) [7, 8, 18]. One trial did not define good functional outcomes, but only had 1 patient discharged whose CPC was 1 [10]. All other patients had a CPC score greater than 3.

**Table 2:** The GRADE approach was used to assess the certainty of evidence

Certainty assessment							No. of patients		Effect		Certainty	Importance
No. of studies	Study design	Risk of bias	Inconsistency	Indirectness	Imprecision	Other considerations	[Corticosteroids]	[Placebo]	Relative (95% CI)	Absolute (95% CI)		
*Mortality*												
8	Randomised trials	Serious^a^	Serious^b^	Not serious	Serious^c^	None	437/920 (47.5%)	548/946 (57.9%)	RR 0.96 (0.90 to 1.02)	23 more per 1000 (from 58 fewer to 12 more)	⨁◯ Very low	Critical
*Return of Spontaneous Circulation*												
4	Randomised trials	Not serious	Not serious	Not serious	Serious^d^	None	257/440 (58.4%)	210/479 (43.8%)	RR 1.32 (1.18 to 1.47)	140 more per 1000 (from 79 to 206 more)	⨁⨁⨁◯ Moderate	Critical
*SURVIVAL WITH GOOD FUNCTIONAL OUTCOME*												
7	Randomised trials	Serious^a^	Serious^b^	Not serious	Serious^c^	None	49/495 (9.9%)	37/534 (6.9%)	RR 1.49 (0.87 to 2.54)	34 more per 1000 (from 9 fewer to 107 more)	⨁⨁◯◯ Low	Critical
*Ventilator Associated Pneumonia*												
2	Randomised trials	Not serious	Not serious	Not serious	Very serious^e^	None	33/178 (18.5%)	29/190 (15.3%)	RR 1.21 (0.77 to 1.90)	32 more per 1000 (from 35 fewer to 137 more)	⨁⨁◯◯ Low	Critical
*Bleeding*												
2	Randomised trials	Serious^f^	Not serious	Serious^g^	Very serious^e^	None	6/155 (3.9%)	3/163 (1.8%)	RR 2.04 (0.53 to 7.84)	19 more per 1000 (from 9 fewer to 126 more)	⨁◯◯◯ Very low	Critical
*Peritonitis*												
2	Randomised trials	Not serious	Not serious	Serious^h^	Very serious^e^	None	3/178 (1.7%)	4/190 (2.1%)	RR 0.82 (0.18 to 3.66)	4 fewer per 1000 (from 17 fewer to 56 more)	⨁◯◯◯ Very low	Important
*Renal Failure*												
2	Randomised trials	Not serious	Not serious	Not serious	Serious^c^	None	24/121 (19.8%)	2/76 (2.6%)	RR 1.29 (0.84 to 1.99)	8 more per 1000 (from 4 fewer to 26 more)	⨁⨁◯◯ Low	Important

*CI* confidence interval, *RR* risk ratio

^a^High Risk of Bias in majority of studies assessing Mortality as an outcome

^b^Important inconsistency in effect with high Isquared and important variation upon visual inspection of the forest plot

^c^Wide confidence intervals with the upper end failing to exclude the possibility of harm

^d^Despite precise 95% CI, number of events below optimal information size contributing to imprecision

^e^Very wide confidence intervals with the upper end failing to exclude the possibility of harm

^f^High Risk of Bias in majority of included studies assessing Bleeding as an outcome

^g^Variation in importance of Bleeding between corticosteroid and control groups

^h^Variation in importance of Peritonitis between corticosteroid and control groups

Corticosteroids may decrease the risk of ventilator associated pneumonia (RR 0.71, 95% CI 0.46–1.09, I2 0%, low certainty), may increase renal failure (RR 1.29, 95% CI 0.84–1.99, I2 0%, low certainty), and have an uncertain effect on bleeding (RR 2.04, 95% CI 0.53–7.84, I2 0%, very low certainty) and peritonitis (RR 10.54, 95% CI 2.99–37.19, I2 0%, very low certainty). See Additional file 1: Appendix 8 for forest plots of all reported outcomes.

Due to lack of sufficient trial level information, the only planned subgroup analysis that we were able to perform was comparing IHCA to OHCA for the outcomes of mortality and survival with good functional outcome (see Additional file 1: Appendix 9 for subgroup analysis forest plots). There was no evidence of effect modification by arrest setting for either of these outcomes of interest.

## Discussion

This systematic review and meta-analysis demonstrates that intravenous corticosteroids administered in the setting of cardiac arrest have an uncertain effect on the risk of mortality, while probably increasing the frequency of ROSC’ and survival with good functional outcome. Certainty related to data on mortality was very low, limited by inconsistency and imprecision. Corticosteroids may increase complications such as ventilator associated pneumonia and renal failure, and they have uncertain effect on bleeding and peritonitis. However, the pooled evidence examining these outcomes was sparse and imprecision contributed to low or very low certainty of evidence.

Previously published systematic reviews and meta-analyses assessing corticosteroids post-cardiac arrest have shown variable and inconclusive results [23–25]. One review found that corticosteroids were associated with increased ROSC and survival to discharge, but retrospective observational studies and randomized controlled trials were pooled in their analysis, an approach that is discouraged by the Cochrane working group [24]. Another meta-analysis, including only RCTs, did not perform quantitative analysis due to insufficient data and instead only provided a narrative summary [25]. A more recent review focused only on IHCA found improvements in neurologic outcomes and survival to hospital discharge with corticosteroids, consistent with our findings [23]. Compared to previous reviews, this report includes the most RCTs and the largest number of patients, thereby providing important precision around key outcomes of interest.

The finding that corticosteroids probably increase ROSC with an uncertain effect on mortality is interesting. Examining the pooled point estimate for mortality and the 95% confidence intervals, the uncertainty does not suggest no effect; rather, the pooled estimate (RR 0.96) is actually consistent with the other outcomes of ROSC and good neurologic recovery; however, limitations in GRADE domains of inconsistency and imprecision led to very low certainty evidence in this outcome. We would be cautious about an intervention that increases ROSC without a clear mortality benefit; however, the possible improvement in survival with good functional outcome with corticosteroids is hopeful. The low certainty evidence for survival with good functional outcome, rated down for inconsistency and imprecision, should provide some caution, and further research is warranted for clarification. Survival with good functional outcome is an outcome that can be challenging to adjudicate given different evaluation time points and issues with loss to follow-up.

Despite a number of RCTs examining the role of corticosteroids in cardiac arrest, there was no standard regimen and variable administration schedules were used amongst the included trials. It is possible that differences in steroid type, dosage, administration timeline, and combination with other drugs (e.g., vasopressin) contributed to the statistical heterogeneity observed in this meta-analysis. This was appropriately accounted for in the GRADE certainty ratings but does contribute to ongoing uncertainty. However, meta-analyses of corticosteroids in other inflammatory conditions (e.g., sepsis and ARDS) have not demonstrated effect modification based on corticosteroid molecule or dose [26, 27]. Further high-quality RCTs assessing the effects of corticosteroids in patients post-cardiac arrest need to be completed to further examine these important considerations.

This review has several strengths. We performed a comprehensive literature search that included recently published trials, undertook dual and independent screening and data abstraction, adhered to our pre-registered protocol, and assessed certainty of outcomes using the GRADE approach. This study also has improved generalizability compared to previous published meta-analyses with the inclusion of IHCA and OHCA patients. This review is the most comprehensive and inclusive to date including data from 2213 patients as compared to the most recently published MA addressing this topic which evaluated data from four RCTs totaling 499 patients [23]. We have included the Andersen study, published in 2021, which enrolled 501 patients [18] and contributes over a quarter of the total patients, increasing the precision in findings and the certainty for overall findings. Additionally, we are the only MA to date to include the Rafiei study, published in 2022 which enrolled 347 patients [21].

This review has several limitations. There was insufficient trial level data to perform most of the planned subgroup analyses. Also, the majority of included RCTs had a high risk of bias and this contributed to low or very low certainty of data of most outcomes of interest. There was also important clinical heterogeneity amongst included studies including cardiac versus noncardiac cause for cardiac arrest, timing and prevalence of bystander CPR, witnessed versus unwitnessed arrest, use of co-interventions such as vasopressin, and steroid type, dose, and timing.

## Conclusion

In patients during or after cardiac arrest, corticosteroids have an uncertain effect on mortality but probably increase ROSC and may increase the likelihood of survival with good functional outcome at hospital discharge. Corticosteroids may decrease ventilator associated pneumonia, may increase renal failure and have an uncertain effect on bleeding and peritonitis. However, the pooled evidence examining these outcomes was sparse and imprecision contributed to low or very low certainty of evidence.

## Supplementary Information

**Additional file 1**. Supplemental information.

## Data Availability

The data that support the findings of this study are openly available in databases CINAHL, EMBASE, LILACS, MEDLINE, Web of Science, CENTRAL, ClinicalTrails.gov, and ICTRP.

## Abbreviations

CI: Confidence interval
CPR: Cardio Pulmonary Resuscitation
CPC: Glasgow Pittsburgh Cerebral Performance Category
ICU: Intensive care unit
IHCA: In-hospital cardiac arrest
OHCA: Out-of-hospital cardiac arrest
RCT: Randomized controlled trial
RR: Relative risk
ROB: Risk of Bias
ROSC: Return of spontaneous circulation
