# Supplementary material for: Efficacy and safety of corticosteroids in cardiac arrest: a systematic review, meta-analysis and trial sequential analysis of randomized control trials
Source: Crit Care. 2023 Jan 11;27:12. doi: 10.1186/s13054-022-04297-2 (PMC9835217; doi:10.1186/s13054-022-04297-2)
Supplement: Supplementary file 1 — Additional file 1. Supplemental information. [file 13054_2022_4297_MOESM1_ESM.docx]

**Supplementary Appendix**

**Supplement to: Jeremy Penn, Will Douglas, Jeffrey Curran, Dipayan Chaudhuri, Joanna Dionne, Shannon Fernando, David Granton, Rebecca Mathew; Bram Rochwerg Efficacy and Assessing Efficacy and Safety of Corticosteroids in Cardiac Arrest Patients: A Systematic Review and Meta-Analysis of Randomized Control Trials**

Table of Contents

[**Appendix 1. EMBASE, MEDLINE, PUBMED Search Strategy** 2](#_Toc121338724)

[**Appendix 2. COCHRANE CENTRAL Search Strategy** 5](#_Toc121338725)

[**Appendix 3. CLINICALTRIALS.gov Search Strategy** 5](#_Toc121338726)

[**Appendix 4. LJILACS Search Strategy** 6](#_Toc121338727)

[**Appendix 5. Web of Science** 6](#_Toc121338728)

[**Appendix 6. WHO International Clinical Trials Registry Platform (ICTRP) Search Strategy** 16](#_Toc121338729)

[**Appendix 7. Risk of Bias Table** 17](#_Toc121338730)

[**Appendix 8. Forest Plot for All Outcomes** 17](#_Toc121338731)

[**Supplement Figure 1. Forest plot. Mortality closest to 28 days:** 17](#_Toc121338732)

[**Supplement Figure 2. Forest plot. Return of Spontaneous Circulation:** 17](#_Toc121338734)

[**Supplement Figure 3. Forest plot. Survival with Good Functional Outcome:** 18](#_Toc121338735)

[**Supplement Figure 4. Forest plot. Renal Failure:** 18](#_Toc121338736)

[**Supplement Figure 5. Forest plot. Peritonitis:** 18](#_Toc121338737)

[**Supplement Figure 6. Forest plot. Bleeding:** 19](#_Toc121338738)

[**Supplement Figure 7. Forest plot. Ventilator Assisted Pneumonia:** 19](#_Toc121338739)

[**Appendix 9. Subgroup Analysis Forest Plots** 19](#_Toc121338740)

[**Supplement Figure 1. Forest plot. Mortality:** 19](#_Toc121338741)

[**Supplement Figure 2. Forest plot. Survival with Good Functional Outcome:** 20](#_Toc121338742)

[**Appendix 10. Trail Sequential Analysis (TSA)** 21](#_Toc121338743)

[**Appendix 11. PRISMA 2020 Table** 22](#_Toc121338744)

**Appendix 1. EMBASE, MEDLINE, PUBMED Search Strategy**

**Embase <1974 to 2020 November 12>, and OVID Medline Epub Ahead of Print, In-Process & Other Non-Indexed Citations, Ovid MEDLINE(R) Daily and Ovid MEDLINE(R) 1946 to Present**

1. Aldosterone/ or (aldosterone or Aldocorten or Aldocortene or Aldocortin or Aldosterona or Aldosterone or Aldosteronum or Electrocortin or Elektrocortin or Reichstein X).ti,ab,kw.

2. exp Hydrocortisone/ or (hydrocortisone or Acticort or Aeroseb-HC or AI3-25006 or Ala-Cort or Ala-Scalp or Alacort or Algicirtis or Amberin or Anflam or Anti-inflammatory hormone or Anucort or Anucort-HC or Anusol HC or Aquacort or Aquanil HC or Balneol-HC or Barseb HC or Basan-Corti or Beta-HC or CCRIS 5854 or Cetacort or Clear aid or Cleiton or Cobadex or Colocort or Compound F or Cort-Dome or Cortanal or Cortef or Cortenema or Cortesal or Corticreme or Cortifan or Cortiment or Cortisol or Cortisol alcohol or Cortisolonum or Cortisporin Otico or Cortispray or Cortolotion or Cortonema or Cortoxide or Cortril or Cremesone or Cremicort-H or Cutisol or Delacort or Derm-Aid or Dermacort or Dermaspray or Dermil or Dermocortal or Dermolate or Dihydrocostisone or Dioderm or Dome-cort or Domolene-HC or Efcorbin or Efcortelan or EINECS or Eldecort or Eldercort or Epicort or Epiderm or Esiderm or Evacort or Ficortril or Flexicort or Foille Insetti or Genacort or Glycort or Gyno-Cortisone or H-Cort or Heb Cort or Heb-Cort or HI-Cor or Hidalone or Hidro-colisona or Hidrocortisona or Hidrocortisona or HSDB 3339 or Hycort or Hycortol or Hycortole or Hydrasson or Hydro-Adreson or Hydro-Colisona or Hydro-RX or Hydrocorticosterone or Hydrocortisonum or Hydrocortisyl or Hydrocortone or Hydroxycortisone or Hytisone or Hytone or Idrocortisone or Incortin-H or Incortin-hydrogen or Kendall's compound F or Komed HC or Kyypakkaus or Lacticare-HC or Lactisona or Lubricort or Maintasone or Meusicort or Mildison or Milliderm or Neosporin-H Ear or Nogenic HC or NSC 10483 or Nutracort or Optef or Otosone-F or Penecort or Permicort or Polcort H or Preparation H Hydrocortisone or Prevex HC or Proctocort or Proctofoam or Proctosol-HC or Proctozone HC or Rectasol-HC or Rectoid or Reichstein's substance M or Remederm HC or Sanatison or Scalp-Cort or Scalpicin Capilar or Schericur or Scheroson F or Sigmacort or Signef or Stie-Cort or Stiefcorcil or Synacort or Systral Hydrocort or Tarcortin or Texacort or Timocort or Transderma H or Traumaide or Uniderm).ti,ab,kw.

3. Cortisone/ or (cortisone or Adrenalex or Andreson or Compound E or Cortisal or Cortisate or Cortisona or Cortisone or Cortisonum or Cortistal or Cortivite or Cortogen or Cortone or Reichstein Fa or compound F).ti,ab,kw.

4. exp Corticosterone/ or (corticosterone or Compound B).ti,ab,kw.

5. exp Pregnenolone/ or (pregnenolone or Arthenolone or Bina-Skin or Enelone or Natolone or Pregneninolone or Pregnenolona or Pregnenolonum or Pregnetan or Pregneton or Pregnolon or Prenolon or Regnosone or Skinostelon).ti,ab,kw.

6. progesterone/ or (progesterone or prometrium or Utrogestan or crinone or Endometrin or Progesteron or cyclogest or prochieve or progest or Progesterona or Proluton or Corpus Luteum Hormones or BHR-100 or Pregnenediones).ti,ab,kw.

7. Clobetasol/ or (clobetason or Clobetasolum or Clobetasol).ti,ab,kw.

8. exp Dexamethasone/ or (dexamethasone or BB 1101 or Decadron or Hexadrol or Alin or Fortecortin or Dexameth or Dexone or Hexadecadrol or Ozurdex or Deronil or Desamethasone or Baycuten or Aacidexam or Spersadex or Dexacortal or Gammacorten or Visumetazone or Dectancyl or Adexone or Alba-Dex or Cortidexason or Decacort or Decadrol or Desmeton or Loverine or Millicorten or Orgadrone or Aknichthol Dexa or auricularum or Auxiloson or Cortisumman or Decalix or Decameth or Decasone or Dekacort or Deltafluorene or Dexa-Mamallet or Dexafluorene or Dexalocal or Dexamecortin or Dexamethasonum or Dexamonozon or DexaposOR Desinoral or Fluorodelta or Lokalison-F or Methylfluorprednisolone or Mymethasone or Amplidermis or Anemul mono or Dexa-Rhinosan or Dexa-Scheroson or Dexa-sine or Dexacortin or Dexafarma or Dinormon or Baycadron or Maxidex or Aeroseb-Dex or Dextenza or Dexasone or Dexpak or Deca or Decaspray or Dexycu or Oradexon or Dexametasona or Corson or Dezone or Soludecadron or Solurex or disaimisong or voren or Cebedex or Dalalone or Decaject or Dekasol or Dexacen or Trabit).ti,ab,kw.

9. Desoximetasone/ or (desoximetasone or topicort or Topicorte).ti,ab,kw.

10. Difluprednate/ or (difluprednate or Durezol or Epitopic).ti,ab,kw.

11. Difluocortolone/ or difluocortolone.ti,ab,kw.

12. Fludrocortisone/ or (fludrocortisone or Florinef or Astonin or Fludrocortison or alpha-Fluorohydrocortisone or FCOL).ti,ab,kw.

13. exp Fluticasone/ or (fluticasone or Flovent or flixotide or Flonase or Veramyst or flixonase or Arnuity or Cutivate or Fluticason).ti,ab,kw.

14. Flucinolone/ or (fluocinolone or Retisert or Iluvien or Synalar).ti,ab,kw.

15. Fluticasone furoate/ or (fluticasone furoate or fluticasone or Flovent or flixotide or Flonase or Veramyst or flixonase or Arnuity or Cutivate or Fluticason).ti,ab,kw.

16. Halometasone/ or (halometasone or Halometasona or Halometasonum or Sicorten).ti,ab,kw.

17. Meprednisone/ or (meprednisone or Betalone or Betanisona or Betapar or Bitanisone or Deltacortene or Deltisona or lepicortin-beta or Meprednisona or Meprednisonum or Methylprednisone).ti,ab,kw.

18. exp Methylprednisolone/ or (methylprednisolone or Prednisolone or Methylprednisolone Acetate or Medrol or Solu-Medrol or Depo-Medrol or Pred Forte or Urbason or medrone or Predate or Wyacort or Delta-F or Duralone or Medrate or Omnipred or Adlone or Caberdelta M or Depmedalone or Depo Moderin or Depo-Nisolone or Emmetipi or Esametone or Decortin H or Firmacort or Medlone or Mega-Star or Meprolone or Methylprednisolonum or Metilbetasone Solubile or Metrocort or Metypresol or Metysolon or orapred or Predni-M-Tablinen or Prednilen or Radilem or Sieropresol or Solpredone or Summicort or Depo-Medrone or A-MethaPred or prelone or Aprednislon or pediapred or deltacortril or Hostacortin H or Di-Adreson-F or Adnisolone or Capsoid or Cortalone or Cortisolone or Estilsona or Panafcortelone or Sterane or Hydrocortancyl or D-Med or Delta-Cortef or Econopred or Methylprednisolon or Dacortin H or Decaprednil or Delta-Diona or Delta-Phoricol or delta1-dehydro-hydrocortisone or Deltahydrocortisone or Deltasolone or Deltidrosol or Dhasolone or Dontisolon D or Fisopred or Frisolona or Gupisone or Hydeltra or Hydeltrasol or Klismacort or Kuhlprednon or Lenisolone or Lepi-Cortinolo or Linola-H N or Linola-H-Fett N or Longiprednil or metacortandralone or Meti Derm or Meticortelone or Opredsone or Precortisyl or Pred-Clysma or Predeltilone or Predni-Coelin or Predni-Helvacort or Prednicortelone or Prednisolonum or Prenilone or Solu Moderin or Pred Mild or Predonine or Flo-Pred or Metypred or prednisol or Pred Fort or Asmacortone or Cryosolona or Medralone or MILLIPRED or Prednilem or Medicort or Prednihexal or Prednesol or Prednisolona or HybriSil or Metilprednisolona or Prednefrin SF or predsol).ti,ab,kw.

19. exp Mometasone Furoate/ or (mometasone furoate or Nasonex or Propel or Sch 32088 or Asmanex or Elocon or Sinuva or Mometason).ti,ab,kw.

20. Paramethasone/ or (paramethasone or Alondra or Cassenne or Cortiden or Dillar or Flumethone or Metilar or Parametasona or Parametasone or Paramethasone or Paramethasonum or Paramezone).ti,ab,kw.

21. Prednisone/ or (prednisone or Deltasone or Decortin or Deltra or Orasone or Meticorten or Cortancyl or deltacortene or Prednicen-M or Dacortin or Adasone or delta-Cortisone or Prednisonum or Decorton or metacortandracin or Panasol or Dehydrocortisone or Predicor or Decortisyl or Delta 1-Cortisone or Delta-Dome or Paracort or deltadehydrocortisone or Ofisolona or Panafcort or Predicorten or Prednicort or Prednidib or Prednilonga or Prednitone or Econosone or Predniment or Promifen or Servisone or Deltison or Lisacort or Meprosona-F or Rayos or sterapred or Liquid Pred or Prednicot or Sone or Cortan or Prednisona or Rectodelt or Predeltin).ti,ab,kw.

22. exp Prednisolone/ or (prednisolone or Methylprednisolone or Methylprednisolone Acetate or Medrol or Solu-Medrol or Depo-Medrol or Pred Forte or Urbason or medrone or Predate or Wyacort or Delta-F or Duralone or Medrate or Omnipred or Adlone or Caberdelta M or Depmedalone or Depo Moderin or Depo-Nisolone or Emmetipi or Esametone or Decortin H or Firmacort or Medlone 21 or Mega-Star or Meprolone or Methylprednisolonum or Metilbetasone Solubile or Metrocort or Metypresol or Metysolon or orapred or Predni-M-Tablinen or Prednilen or Radilem or Sieropresol or Solpredone or Summicort or Depo-Medrone or A-MethaPred or prelone or Aprednislon or pediapred or deltacortril or Hostacortin H or Di-Adreson-F or Adnisolone or Capsoid or Cortalone or Cortisolone or Estilsona or Panafcortelone or Sterane or Hydrocortancyl or D-Med or Delta-Cortef or Econopred or Methylprednisolon or Dacortin H or Decaprednil or Delta-Diona or Delta-Phoricol or delta1-dehydro-hydrocortisone or Deltahydrocortisone or Deltasolone or Deltidrosol or Dhasolone or Dontisolon D or Fisopred or Frisolona or Gupisone or Hydeltra or Hydeltrasol or Klismacort or Kuhlprednon or Lenisolone or Lepi-Cortinolo or Linola-H N or Linola-H-Fett N or Longiprednil or metacortandralone or Meti Derm or Meticortelone or Opredsone or Precortisyl or Pred-Clysma or Predeltilone or Predni-Coelin or Predni-Helvacort or Prednicortelone or Prednisolonum or Prenilone or Solu Moderin or Pred Mild or Predonine or Flo-Pred or Metypred or prednisol or Pred Fort or Asmacortone or Cryosolona or Medralone or MILLIPRED or Prednilem or Medicort or Prednihexal or Prednesol or Prednisolona or HybriSil or Metilprednisolona or Prednefrin SF or predsol).ti,ab,kw.

23. Prednicarbate/ or (prednicarbate or Batmen or dermatop).ti,ab,kw.

24. Tixocortol/ or (Tixocortol or Tixocortolum).ti,ab,kw.

25. exp Triamcinolone/ or (triamcinolone or TAC or Kenalog or kenacort or Zilretta or nasacort or Triesence or Volon or Aristocort or triam or Aristospan or Azmacort or Cinonide or Tricort 40 or Tri-Nasal or Triamcinolona or Trilone or Tristoject).ti,ab,kw.

26. exp Adrenal Cortex Hormones/ or exp corticosteroid/ or (corticosteroid or corticosteroids).ti,ab,kw.

27. or/1-26

28. exp Heart Arrest/ or (heart arrest or heart standstill).ti,ab,kw.

29. ((cardiac or cardiopulmonary or circulation or circulatory or heart) adj arrest).ti,ab,kw.

30. asystole.mp. or asystoles.ti,ab,kw. [mp=ti, ab, hw, tn, ot, dm, mf, dv, kw, fx, dq, nm, kf, ox, px, rx, ui, sy]

31. sudden cardiac death/ or sudden cardiac death.ti,ab,kw.

32. exp sudden death/

33. karoshi?death?.ti,ab,kw. or occupational sudden death/

34. cardiopulmonary resuscitation/ or (cardio?pulmonary resuscitation or mouth-to-mouth resuscitation).ti,ab,kw.

35. shock reversal.ti,ab,kw.

36. exp cardiogenic shock/

37. or/28-36

38. Randomized Controlled Trial.pt.

39. Pragmatic Clinical Trial.pt.

40. exp Randomized Controlled Trials as Topic/

41. "Randomized Controlled Trial (topic)"/

42. Randomized Controlled Trial/

43. Randomization/

44. Random Allocation/

45. Double-Blind Method/

46. Double Blind Procedure/

47. Double-Blind Studies/

48. Single-Blind Method/

49. Single Blind Procedure/

50. Single-Blind Studies/

51. Placebos/

52. Placebo/

53. (random* or sham or placebo*).ti,ab,hw,kw.

54. ((singl* or doubl*) adj (blind* or dumm* or mask*)).ti,ab,hw,kw.

55. ((tripl* or trebl*) adj (blind* or dumm* or mask*)).ti,ab,hw,kw.

56. or/38-55

57. exp animals/

58. exp animal experimentation/ or exp animal experiment/

59. exp models animal/

60. nonhuman/

61. exp vertebrate/ or exp vertebrates/

62. 57 or 58 or 59 or 60 or 61

63. exp humans/

64. exp human experimentation/ or exp human experiment/

65. 63 or 64

66. 62 not 65

67. (27 and 37 and 56) not 66

68. 67 use ppez

69. 67 use oemezd

70. remove duplicates from 67

**Appendix 2. COCHRANE CENTRAL Search Strategy**

**COCHRANE Controlled Clinical Trials Registry (*The Cochrane Library* Issue 9, 2019)**

ID Search Hits

#1 (corticosteroid or corticosteroids or steroid or steroids or aldosterone or hydrocortisone or cortisone or corticosterone or pregnenolone or progesterone or clobetasol or dexamethasone or desoximetasone or difluprednate or difluocortolone or fludrocortisone or fluticasone or flucinolone or fluticasone or halometasone or meprednisone or mometasone or paramethasone or prednisone or prednisolone or prednicarbate or tixocortol or triamcinolone):ti,ab,kw 82902

#2 (heart arrest OR heart standstill OR asystole OR asystoles OR sudden cardiac death OR sudden death OR cardiopumonary resuscitation OR shock reversal OR cardiogenic shock):ti,ab,kw 8512

#3 MeSH descriptor: [Heart Arrest] explode all trees 1943

#4 MeSH descriptor: [Death, Sudden, Cardiac] explode all trees 624

#5 MeSH descriptor: [Death, Sudden] explode all trees 851

#6 MeSH descriptor: [Cardiopulmonary Resuscitation] explode all trees 1075

#7 MeSH descriptor: [Shock, Cardiogenic] explode all trees 257

#8 #2 or #3 or #4 or #5 or #6 or #7 9136

#9 #1 and #8 334

334 clinical trials

**Appendix 3. CLINICALTRIALS.gov Search Strategy**

**Clinical Trials.gov**

Advanced search, no date limit applied

Condition or disease: heart arrest OR heart standstill OR asystole OR asystoles OR sudden cardiac death OR sudden death OR cardiopumonary resuscitation OR shock reversal OR cardiogenic shock

AND

Other terms: "corticosteroid" OR "steroid" OR "hydrocortisone" OR “cortisone” OR “corticosterone” OR “pregnenolone” OR “progesterone” OR “clobetasol” OR “dexamethasone” OR “desoximetasone” OR “difluprednate” OR “prednisone” OR “prednisolone” OR “aldosterone”

(due to the limit of terms, this second batch of terms was searched additional to the paragraph above, but yielded no new results)

OR “difluocortolone” OR “fludrocortisone” OR “fluticasone” OR “flucinolone” OR “fluticasone” OR “halometasone” OR “meprednisone” OR “mometasone” OR “paramethasone” OR “prednicarbate” OR “tixocortol” OR “triamcinolone”

Study type: “Interventional studies (clinical trials)”

10 studies

**Appendix 4. LJILACS Search Strategy**

**Advanced search**

Words: heart arrest OR heart standstill OR asystole OR asystoles OR sudden cardiac death OR sudden death OR cardiopumonary resuscitation OR shock reversal OR cardiogenic shock

AND

Words: corticosteroid OR corticosteroids OR steroid OR steroids OR aldosterone OR hydrocortisone OR cortisone OR corticosterone OR pregnenolone OR progesterone OR clobetasol OR dexamethasone OR desoximetasone OR difluprednate OR difluocortolone OR fludrocortisone OR fluticasone OR flucinolone OR fluticasone OR halometasone OR meprednisone OR mometasone OR paramethasone OR prednisone OR prednisolone OR prednicarbate OR tixocortol OR triamcinolone

6 citations

**Appendix 5. Web of Science**

**Web of Science (core collection, 1976-2020)**

| # 38 | [1,304](https://apps-webofknowledge-com.libaccess.lib.mcmaster.ca/summary.do?product=WOS&doc=1&qid=55&SID=8BznjzuJYXZp9nJ79Pv&search_mode=CombineSearches&update_back2search_link_param=yes) | #37 AND #27 |
| --- | --- | --- |
|  |  | *Indexes=SCI-EXPANDED, SSCI, A&HCI, CPCI-S, CPCI-SSH, BKCI-S, BKCI-SSH, ESCI, CCR-EXPANDED, IC Timespan=All years* |
| # 37 | [130,808](https://apps-webofknowledge-com.libaccess.lib.mcmaster.ca/summary.do?product=WOS&doc=1&qid=54&SID=8BznjzuJYXZp9nJ79Pv&search_mode=CombineSearches&update_back2search_link_param=yes) | #36 OR #35 OR #34 OR #33 OR #32 OR #31 OR #30 OR #29 OR #28 |
|  |  | *Indexes=SCI-EXPANDED, SSCI, A&HCI, CPCI-S, CPCI-SSH, BKCI-S, BKCI-SSH, ESCI, CCR-EXPANDED, IC Timespan=All years* |
| # 36 | [12,102](https://apps-webofknowledge-com.libaccess.lib.mcmaster.ca/summary.do?product=WOS&doc=1&qid=53&SID=8BznjzuJYXZp9nJ79Pv&search_mode=AdvancedSearch&update_back2search_link_param=yes) | KP=(cardiogenic shock) OR TI=(cardiogenic shock) OR AB=(cardiogenic shock) |
|  |  | *Indexes=SCI-EXPANDED, SSCI, A&HCI, CPCI-S, CPCI-SSH, BKCI-S, BKCI-SSH, ESCI, CCR-EXPANDED, IC Timespan=All years* |
| # 35 | [1,274](https://apps-webofknowledge-com.libaccess.lib.mcmaster.ca/summary.do?product=WOS&doc=1&qid=52&SID=8BznjzuJYXZp9nJ79Pv&search_mode=AdvancedSearch&update_back2search_link_param=yes) | KP=(shock reversal) OR TI=(shock reversal) or AB=(shock reversal) |
|  |  | *Indexes=SCI-EXPANDED, SSCI, A&HCI, CPCI-S, CPCI-SSH, BKCI-S, BKCI-SSH, ESCI, CCR-EXPANDED, IC Timespan=All years* |
| # 34 | [22,704](https://apps-webofknowledge-com.libaccess.lib.mcmaster.ca/summary.do?product=WOS&doc=1&qid=51&SID=8BznjzuJYXZp9nJ79Pv&search_mode=AdvancedSearch&update_back2search_link_param=yes) | KP=(cardiopulmonary resuscitation or mouth-to-mouth resuscitation) OR TI=(cardiopulmonary resuscitation or mouth-to-mouth resuscitation) OR AB=(cardiopulmonary resuscitation or mouth-to-mouth resuscitation) |
|  |  | *Indexes=SCI-EXPANDED, SSCI, A&HCI, CPCI-S, CPCI-SSH, BKCI-S, BKCI-SSH, ESCI, CCR-EXPANDED, IC Timespan=All years* |
| # 33 | [28](https://apps-webofknowledge-com.libaccess.lib.mcmaster.ca/summary.do?product=WOS&doc=1&qid=50&SID=8BznjzuJYXZp9nJ79Pv&search_mode=AdvancedSearch&update_back2search_link_param=yes) | KP=(Karoshi death) or TI=(Karoshi death) or AB=(karoshi death) |
|  |  | *Indexes=SCI-EXPANDED, SSCI, A&HCI, CPCI-S, CPCI-SSH, BKCI-S, BKCI-SSH, ESCI, CCR-EXPANDED, IC Timespan=All years* |
| # 32 | [56,289](https://apps-webofknowledge-com.libaccess.lib.mcmaster.ca/summary.do?product=WOS&doc=1&qid=49&SID=8BznjzuJYXZp9nJ79Pv&search_mode=AdvancedSearch&update_back2search_link_param=yes) | KP=(sudden death) OR TI=(sudden death) or AB=(Sudden death) |
|  |  | *Indexes=SCI-EXPANDED, SSCI, A&HCI, CPCI-S, CPCI-SSH, BKCI-S, BKCI-SSH, ESCI, CCR-EXPANDED, IC Timespan=All years* |
| # 31 | [27,129](https://apps-webofknowledge-com.libaccess.lib.mcmaster.ca/summary.do?product=WOS&doc=1&qid=48&SID=8BznjzuJYXZp9nJ79Pv&search_mode=AdvancedSearch&update_back2search_link_param=yes) | KP=(sudden cardiac death) or TI=(sudden cardiac death) or AB=(sudden cardiac death) |
|  |  | *Indexes=SCI-EXPANDED, SSCI, A&HCI, CPCI-S, CPCI-SSH, BKCI-S, BKCI-SSH, ESCI, CCR-EXPANDED, IC Timespan=All years* |
| # 30 | [3,320](https://apps-webofknowledge-com.libaccess.lib.mcmaster.ca/summary.do?product=WOS&doc=1&qid=46&SID=8BznjzuJYXZp9nJ79Pv&search_mode=AdvancedSearch&update_back2search_link_param=yes) | KP=(asystole or asystoles) OR TI=(asystole or asystoles) or AB=(asystole or asystoles) |
|  |  | *Indexes=SCI-EXPANDED, SSCI, A&HCI, CPCI-S, CPCI-SSH, BKCI-S, BKCI-SSH, ESCI, CCR-EXPANDED, IC Timespan=All years* |
| # 29 | [55,427](https://apps-webofknowledge-com.libaccess.lib.mcmaster.ca/summary.do?product=WOS&doc=1&qid=45&SID=8BznjzuJYXZp9nJ79Pv&search_mode=AdvancedSearch&update_back2search_link_param=yes) | KP=(cardiac arrest or cardiopulmonary arrest or circulation arrest or circulatory arrest or heart arrest) OR TI=(cardiac arrest or cardiopulmonary arrest or circulation arrest or circulatory arrest or heart arrest) OR AB=(cardiac arrest or cardiopulmonary arrest or circulation arrest or circulatory arrest or heart arrest) |
|  |  | *Indexes=SCI-EXPANDED, SSCI, A&HCI, CPCI-S, CPCI-SSH, BKCI-S, BKCI-SSH, ESCI, CCR-EXPANDED, IC Timespan=All years* |
| # 28 | [11,657](https://apps-webofknowledge-com.libaccess.lib.mcmaster.ca/summary.do?product=WOS&doc=1&qid=44&SID=8BznjzuJYXZp9nJ79Pv&search_mode=AdvancedSearch&update_back2search_link_param=yes) | KP=(heart arrest or heart standstill) OR AB=(heart arrest or heart standstill) OR TI=(heart arrest or heart standstill) |
|  |  | *Indexes=SCI-EXPANDED, SSCI, A&HCI, CPCI-S, CPCI-SSH, BKCI-S, BKCI-SSH, ESCI, CCR-EXPANDED, IC Timespan=All years* |
| # 27 | [803,354](https://apps-webofknowledge-com.libaccess.lib.mcmaster.ca/summary.do?product=WOS&doc=1&qid=43&SID=8BznjzuJYXZp9nJ79Pv&search_mode=CombineSearches&update_back2search_link_param=yes) | #26 OR #25 OR #24 OR #23 OR #22 OR #21 OR #20 OR #19 OR #18 OR #17 OR #16 OR #15 OR #14 OR #13 OR #12 OR #11 OR #10 OR #9 OR #8 OR #7 OR #6 OR #5 OR #4 OR #3 OR #2 OR #1 |
|  |  | *Indexes=SCI-EXPANDED, SSCI, A&HCI, CPCI-S, CPCI-SSH, BKCI-S, BKCI-SSH, ESCI, CCR-EXPANDED, IC Timespan=All years* |
| # 26 | [84,555](https://apps-webofknowledge-com.libaccess.lib.mcmaster.ca/summary.do?product=WOS&doc=1&qid=42&SID=8BznjzuJYXZp9nJ79Pv&search_mode=AdvancedSearch&update_back2search_link_param=yes) | AB=(Adrenal Cortex Hormones or corticosteroid or corticosteroids) OR TI=(Adrenal Cortex Hormones or corticosteroid or corticosteroids) |
|  |  | *Indexes=SCI-EXPANDED, SSCI, A&HCI, CPCI-S, CPCI-SSH, BKCI-S, BKCI-SSH, ESCI, CCR-EXPANDED, IC Timespan=All years* |
| # 25 | [21,519](https://apps-webofknowledge-com.libaccess.lib.mcmaster.ca/summary.do?product=WOS&doc=1&qid=40&SID=8BznjzuJYXZp9nJ79Pv&search_mode=AdvancedSearch&update_back2search_link_param=yes) | AB=(triamcinolone or TAC or Kenalog or kenacort or Zilretta or nasacort or Triesence or Volon or Aristocort or triam or Aristospan or Azmacort or Cinonide or Tricort 40 or Tri-Nasal or Triamcinolona or Trilone or Tristoject) OR TI=(triamcinolone or TAC or Kenalog or kenacort or Zilretta or nasacort or Triesence or Volon or Aristocort or triam or Aristospan or Azmacort or Cinonide or Tricort 40 or Tri-Nasal or Triamcinolona or Trilone or Tristoject) |
|  |  | *Indexes=SCI-EXPANDED, SSCI, A&HCI, CPCI-S, CPCI-SSH, BKCI-S, BKCI-SSH, ESCI, CCR-EXPANDED, IC Timespan=All years* |
| # 24 | [133](https://apps-webofknowledge-com.libaccess.lib.mcmaster.ca/summary.do?product=WOS&doc=1&qid=39&SID=8BznjzuJYXZp9nJ79Pv&search_mode=AdvancedSearch&update_back2search_link_param=yes) | AB=(Tixocortol or Tixocortolum) OR TI=(Tixocortol or Tixocortolum) |
|  |  | *Indexes=SCI-EXPANDED, SSCI, A&HCI, CPCI-S, CPCI-SSH, BKCI-S, BKCI-SSH, ESCI, CCR-EXPANDED, IC Timespan=All years* |
| # 23 | [808](https://apps-webofknowledge-com.libaccess.lib.mcmaster.ca/summary.do?product=WOS&doc=1&qid=37&SID=8BznjzuJYXZp9nJ79Pv&search_mode=AdvancedSearch&update_back2search_link_param=yes) | AB=(prednicarbate or Batmen or dermatop) OR TI=(prednicarbate or Batmen or dermatop) |
|  |  | *Indexes=SCI-EXPANDED, SSCI, A&HCI, CPCI-S, CPCI-SSH, BKCI-S, BKCI-SSH, ESCI, CCR-EXPANDED, IC Timespan=All years* |
| # 22 | [47,400](https://apps-webofknowledge-com.libaccess.lib.mcmaster.ca/summary.do?product=WOS&doc=1&qid=35&SID=8BznjzuJYXZp9nJ79Pv&search_mode=AdvancedSearch&update_back2search_link_param=yes) | AB=(prednisolone or Methylprednisolone or Methylprednisolone Acetate or Medrol or Solu-Medrol or Depo-Medrol or Pred Forte or Urbason or medrone or Predate or Wyacort or Delta-F or Duralone or Medrate or Omnipred or Adlone or Caberdelta M or Depmedalone or Depo Moderin or Depo-Nisolone or Emmetipi or Esametone or Decortin H or Firmacort or Medlone 21 or Mega-Star or Meprolone or Methylprednisolonum or Metilbetasone Solubile or Metrocort or Metypresol or Metysolon or orapred or Predni-M-Tablinen or Prednilen or Radilem or Sieropresol or Solpredone or Summicort or Depo-Medrone or A-MethaPred or prelone or Aprednislon or pediapred or deltacortril or Hostacortin H or Di-Adreson-F or Adnisolone or Capsoid or Cortalone or Cortisolone or Estilsona or Panafcortelone or Sterane or Hydrocortancyl or D-Med or Delta-Cortef or Econopred or Methylprednisolon or Dacortin H or Decaprednil or Delta-Diona or Delta-Phoricol or delta1-dehydro-hydrocortisone or Deltahydrocortisone or Deltasolone or Deltidrosol or Dhasolone or Dontisolon D or Fisopred or Frisolona or Gupisone or Hydeltra or Hydeltrasol or Klismacort or Kuhlprednon or Lenisolone or Lepi-Cortinolo or Linola-H N or Linola-H-Fett N or Longiprednil or metacortandralone or Meti Derm or Meticortelone or Opredsone or Precortisyl or Pred-Clysma or Predeltilone or Predni-Coelin or Predni-Helvacort or Prednicortelone or Prednisolonum or Prenilone or Solu Moderin or Pred Mild or Predonine or Flo-Pred or Metypred or prednisol or Pred Fort or Asmacortone or Cryosolona or Medralone or MILLIPRED or Prednilem or Medicort or Prednihexal or Prednesol or Prednisolona or HybriSil or Metilprednisolona or Prednefrin SF or predsol) OR TI=(prednisolone or Methylprednisolone or Methylprednisolone Acetate or Medrol or Solu-Medrol or Depo-Medrol or Pred Forte or Urbason or medrone or Predate or Wyacort or Delta-F or Duralone or Medrate or Omnipred or Adlone or Caberdelta M or Depmedalone or Depo Moderin or Depo-Nisolone or Emmetipi or Esametone or Decortin H or Firmacort or Medlone 21 or Mega-Star or Meprolone or Methylprednisolonum or Metilbetasone Solubile or Metrocort or Metypresol or Metysolon or orapred or Predni-M-Tablinen or Prednilen or Radilem or Sieropresol or Solpredone or Summicort or Depo-Medrone or A-MethaPred or prelone or Aprednislon or pediapred or deltacortril or Hostacortin H or Di-Adreson-F or Adnisolone or Capsoid or Cortalone or Cortisolone or Estilsona or Panafcortelone or Sterane or Hydrocortancyl or D-Med or Delta-Cortef or Econopred or Methylprednisolon or Dacortin H or Decaprednil or Delta-Diona or Delta-Phoricol or delta1-dehydro-hydrocortisone or Deltahydrocortisone or Deltasolone or Deltidrosol or Dhasolone or Dontisolon D or Fisopred or Frisolona or Gupisone or Hydeltra or Hydeltrasol or Klismacort or Kuhlprednon or Lenisolone or Lepi-Cortinolo or Linola-H N or Linola-H-Fett N or Longiprednil or metacortandralone or Meti Derm or Meticortelone or Opredsone or Precortisyl or Pred-Clysma or Predeltilone or Predni-Coelin or Predni-Helvacort or Prednicortelone or Prednisolonum or Prenilone or Solu Moderin or Pred Mild or Predonine or Flo-Pred or Metypred or prednisol or Pred Fort or Asmacortone or Cryosolona or Medralone or MILLIPRED or Prednilem or Medicort or Prednihexal or Prednesol or Prednisolona or HybriSil or Metilprednisolona or Prednefrin SF or predsol) |
|  |  | *Indexes=SCI-EXPANDED, SSCI, A&HCI, CPCI-S, CPCI-SSH, BKCI-S, BKCI-SSH, ESCI, CCR-EXPANDED, IC Timespan=All years* |
| # 21 | [21,177](https://apps-webofknowledge-com.libaccess.lib.mcmaster.ca/summary.do?product=WOS&doc=1&qid=33&SID=8BznjzuJYXZp9nJ79Pv&search_mode=AdvancedSearch&update_back2search_link_param=yes) | AB=(prednisone or Deltasone or Decortin or Deltra or Orasone or Meticorten or Cortancyl or deltacortene or Prednicen-M or Dacortin or Adasone or delta-Cortisone or Prednisonum or Decorton or metacortandracin or Panasol or Dehydrocortisone or Predicor or Decortisyl or Delta 1-Cortisone or Delta-Dome or Paracort or deltadehydrocortisone or Ofisolona or Panafcort or Predicorten or Prednicort or Prednidib or Prednilonga or Prednitone or Econosone or Predniment or Promifen or Servisone or Deltison or Lisacort or Meprosona-F or Rayos or sterapred or Liquid Pred or Prednicot or Sone or Cortan or Prednisona or Rectodelt or Predeltin) OR TI=(prednisone or Deltasone or Decortin or Deltra or Orasone or Meticorten or Cortancyl or deltacortene or Prednicen-M or Dacortin or Adasone or delta-Cortisone or Prednisonum or Decorton or metacortandracin or Panasol or Dehydrocortisone or Predicor or Decortisyl or Delta 1-Cortisone or Delta-Dome or Paracort or deltadehydrocortisone or Ofisolona or Panafcort or Predicorten or Prednicort or Prednidib or Prednilonga or Prednitone or Econosone or Predniment or Promifen or Servisone or Deltison or Lisacort or Meprosona-F or Rayos or sterapred or Liquid Pred or Prednicot or Sone or Cortan or Prednisona or Rectodelt or Predeltin) |
|  |  | *Indexes=SCI-EXPANDED, SSCI, A&HCI, CPCI-S, CPCI-SSH, BKCI-S, BKCI-SSH, ESCI, CCR-EXPANDED, IC Timespan=All years* |
| # 20 | [53](https://apps-webofknowledge-com.libaccess.lib.mcmaster.ca/summary.do?product=WOS&doc=1&qid=31&SID=8BznjzuJYXZp9nJ79Pv&search_mode=AdvancedSearch&update_back2search_link_param=yes) | AB=(paramethasone or Alondra or Cassenne or Cortiden or Dillar or Flumethone or Metilar or Parametasona or Parametasone or Paramethasone or Paramethasonum or Paramezone) OR TI=(paramethasone or Alondra or Cassenne or Cortiden or Dillar or Flumethone or Metilar or Parametasona or Parametasone or Paramethasone or Paramethasonum or Paramezone) |
|  |  | *Indexes=SCI-EXPANDED, SSCI, A&HCI, CPCI-S, CPCI-SSH, BKCI-S, BKCI-SSH, ESCI, CCR-EXPANDED, IC Timespan=All years* |
| # 19 | [18,895](https://apps-webofknowledge-com.libaccess.lib.mcmaster.ca/summary.do?product=WOS&doc=1&qid=29&SID=8BznjzuJYXZp9nJ79Pv&search_mode=AdvancedSearch&update_back2search_link_param=yes) | AB=(mometasone furoate or Nasonex or Propel or Sch 32088 or Asmanex or Elocon or Sinuva or Mometason) OR TI=(mometasone furoate or Nasonex or Propel or Sch 32088 or Asmanex or Elocon or Sinuva or Mometason) |
|  |  | *Indexes=SCI-EXPANDED, SSCI, A&HCI, CPCI-S, CPCI-SSH, BKCI-S, BKCI-SSH, ESCI, CCR-EXPANDED, IC Timespan=All years* |
| # 18 | [47,400](https://apps-webofknowledge-com.libaccess.lib.mcmaster.ca/summary.do?product=WOS&doc=1&qid=27&SID=8BznjzuJYXZp9nJ79Pv&search_mode=AdvancedSearch&update_back2search_link_param=yes) | AB=(methylprednisolone or Prednisolone or Methylprednisolone Acetate or Medrol or Solu-Medrol or Depo-Medrol or Pred Forte or Urbason or medrone or Predate or Wyacort or Delta-F or Duralone or Medrate or Omnipred or Adlone or Caberdelta M or Depmedalone or Depo Moderin or Depo-Nisolone or Emmetipi or Esametone or Decortin H or Firmacort or Medlone or Mega-Star or Meprolone or Methylprednisolonum or Metilbetasone Solubile or Metrocort or Metypresol or Metysolon or orapred or Predni-M-Tablinen or Prednilen or Radilem or Sieropresol or Solpredone or Summicort or Depo-Medrone or A-MethaPred or prelone or Aprednislon or pediapred or deltacortril or Hostacortin H or Di-Adreson-F or Adnisolone or Capsoid or Cortalone or Cortisolone or Estilsona or Panafcortelone or Sterane or Hydrocortancyl or D-Med or Delta-Cortef or Econopred or Methylprednisolon or Dacortin H or Decaprednil or Delta-Diona or Delta-Phoricol or delta1-dehydro-hydrocortisone or Deltahydrocortisone or Deltasolone or Deltidrosol or Dhasolone or Dontisolon D or Fisopred or Frisolona or Gupisone or Hydeltra or Hydeltrasol or Klismacort or Kuhlprednon or Lenisolone or Lepi-Cortinolo or Linola-H N or Linola-H-Fett N or Longiprednil or metacortandralone or Meti Derm or Meticortelone or Opredsone or Precortisyl or Pred-Clysma or Predeltilone or Predni-Coelin or Predni-Helvacort or Prednicortelone or Prednisolonum or Prenilone or Solu Moderin or Pred Mild or Predonine or Flo-Pred or Metypred or prednisol or Pred Fort or Asmacortone or Cryosolona or Medralone or MILLIPRED or Prednilem or Medicort or Prednihexal or Prednesol or Prednisolona or HybriSil or Metilprednisolona or Prednefrin SF or predsol) OR TI=(methylprednisolone or Prednisolone or Methylprednisolone Acetate or Medrol or Solu-Medrol or Depo-Medrol or Pred Forte or Urbason or medrone or Predate or Wyacort or Delta-F or Duralone or Medrate or Omnipred or Adlone or Caberdelta M or Depmedalone or Depo Moderin or Depo-Nisolone or Emmetipi or Esametone or Decortin H or Firmacort or Medlone or Mega-Star or Meprolone or Methylprednisolonum or Metilbetasone Solubile or Metrocort or Metypresol or Metysolon or orapred or Predni-M-Tablinen or Prednilen or Radilem or Sieropresol or Solpredone or Summicort or Depo-Medrone or A-MethaPred or prelone or Aprednislon or pediapred or deltacortril or Hostacortin H or Di-Adreson-F or Adnisolone or Capsoid or Cortalone or Cortisolone or Estilsona or Panafcortelone or Sterane or Hydrocortancyl or D-Med or Delta-Cortef or Econopred or Methylprednisolon or Dacortin H or Decaprednil or Delta-Diona or Delta-Phoricol or delta1-dehydro-hydrocortisone or Deltahydrocortisone or Deltasolone or Deltidrosol or Dhasolone or Dontisolon D or Fisopred or Frisolona or Gupisone or Hydeltra or Hydeltrasol or Klismacort or Kuhlprednon or Lenisolone or Lepi-Cortinolo or Linola-H N or Linola-H-Fett N or Longiprednil or metacortandralone or Meti Derm or Meticortelone or Opredsone or Precortisyl or Pred-Clysma or Predeltilone or Predni-Coelin or Predni-Helvacort or Prednicortelone or Prednisolonum or Prenilone or Solu Moderin or Pred Mild or Predonine or Flo-Pred or Metypred or prednisol or Pred Fort or Asmacortone or Cryosolona or Medralone or MILLIPRED or Prednilem or Medicort or Prednihexal or Prednesol or Prednisolona or HybriSil or Metilprednisolona or Prednefrin SF or predsol) |
|  |  | *Indexes=SCI-EXPANDED, SSCI, A&HCI, CPCI-S, CPCI-SSH, BKCI-S, BKCI-SSH, ESCI, CCR-EXPANDED, IC Timespan=All years* |
| # 17 | [224](https://apps-webofknowledge-com.libaccess.lib.mcmaster.ca/summary.do?product=WOS&doc=1&qid=26&SID=8BznjzuJYXZp9nJ79Pv&search_mode=AdvancedSearch&update_back2search_link_param=yes) | AB=(meprednisone or Betalone or Betanisona or Betapar or Bitanisone or Deltacortene or Deltisona or lepicortin-beta or Meprednisona or Meprednisonum or Methylprednisone) OR TI=(meprednisone or Betalone or Betanisona or Betapar or Bitanisone or Deltacortene or Deltisona or lepicortin-beta or Meprednisona or Meprednisonum or Methylprednisone) |
|  |  | *Indexes=SCI-EXPANDED, SSCI, A&HCI, CPCI-S, CPCI-SSH, BKCI-S, BKCI-SSH, ESCI, CCR-EXPANDED, IC Timespan=All years* |
| # 16 | [36](https://apps-webofknowledge-com.libaccess.lib.mcmaster.ca/summary.do?product=WOS&doc=1&qid=24&SID=8BznjzuJYXZp9nJ79Pv&search_mode=AdvancedSearch&update_back2search_link_param=yes) | AB=(halometasone or Halometasona or Halometasonum or Sicorten) OR TI=(halometasone or Halometasona or Halometasonum or Sicorten) |
|  |  | *Indexes=SCI-EXPANDED, SSCI, A&HCI, CPCI-S, CPCI-SSH, BKCI-S, BKCI-SSH, ESCI, CCR-EXPANDED, IC Timespan=All years* |
| # 15 | [4,901](https://apps-webofknowledge-com.libaccess.lib.mcmaster.ca/summary.do?product=WOS&doc=1&qid=22&SID=8BznjzuJYXZp9nJ79Pv&search_mode=AdvancedSearch&update_back2search_link_param=yes) | AB=(fluticasone furoate or fluticasone or Flovent or flixotide or Flonase or Veramyst or flixonase or Arnuity or Cutivate or Fluticason) OR TI=(fluticasone furoate or fluticasone or Flovent or flixotide or Flonase or Veramyst or flixonase or Arnuity or Cutivate or Fluticason) |
|  |  | *Indexes=SCI-EXPANDED, SSCI, A&HCI, CPCI-S, CPCI-SSH, BKCI-S, BKCI-SSH, ESCI, CCR-EXPANDED, IC Timespan=All years* |
| # 14 | [678](https://apps-webofknowledge-com.libaccess.lib.mcmaster.ca/summary.do?product=WOS&doc=1&qid=21&SID=8BznjzuJYXZp9nJ79Pv&search_mode=AdvancedSearch&update_back2search_link_param=yes) | AB=(fluocinolone or Retisert or Iluvien or Synalar) OR TI=(fluocinolone or Retisert or Iluvien or Synalar) |
|  |  | *Indexes=SCI-EXPANDED, SSCI, A&HCI, CPCI-S, CPCI-SSH, BKCI-S, BKCI-SSH, ESCI, CCR-EXPANDED, IC Timespan=All years* |
| # 13 | [4,901](https://apps-webofknowledge-com.libaccess.lib.mcmaster.ca/summary.do?product=WOS&doc=1&qid=19&SID=8BznjzuJYXZp9nJ79Pv&search_mode=AdvancedSearch&update_back2search_link_param=yes) | AB=(fluticasone or Flovent or flixotide or Flonase or Veramyst or flixonase or Arnuity or Cutivate or Fluticason) OR TI=(fluticasone or Flovent or flixotide or Flonase or Veramyst or flixonase or Arnuity or Cutivate or Fluticason) |
|  |  | *Indexes=SCI-EXPANDED, SSCI, A&HCI, CPCI-S, CPCI-SSH, BKCI-S, BKCI-SSH, ESCI, CCR-EXPANDED, IC Timespan=All years* |
| # 12 | [953](https://apps-webofknowledge-com.libaccess.lib.mcmaster.ca/summary.do?product=WOS&doc=1&qid=18&SID=8BznjzuJYXZp9nJ79Pv&search_mode=AdvancedSearch&update_back2search_link_param=yes) | AB=(fludrocortisone or Florinef or Astonin or Fludrocortison or alpha-Fluorohydrocortisone or FCOL) OR TI=(fludrocortisone or Florinef or Astonin or Fludrocortison or alpha-Fluorohydrocortisone or FCOL) |
|  |  | *Indexes=SCI-EXPANDED, SSCI, A&HCI, CPCI-S, CPCI-SSH, BKCI-S, BKCI-SSH, ESCI, CCR-EXPANDED, IC Timespan=All years* |
| # 11 | [4](https://apps-webofknowledge-com.libaccess.lib.mcmaster.ca/summary.do?product=WOS&doc=1&qid=16&SID=8BznjzuJYXZp9nJ79Pv&search_mode=AdvancedSearch&update_back2search_link_param=yes) | AB=(difluocortolone) or TI=(difluocortolone) |
|  |  | *Indexes=SCI-EXPANDED, SSCI, A&HCI, CPCI-S, CPCI-SSH, BKCI-S, BKCI-SSH, ESCI, CCR-EXPANDED, IC Timespan=All years* |
| # 10 | [786](https://apps-webofknowledge-com.libaccess.lib.mcmaster.ca/summary.do?product=WOS&doc=1&qid=15&SID=8BznjzuJYXZp9nJ79Pv&search_mode=AdvancedSearch&update_back2search_link_param=yes) | AB=(difluprednate or Durezol or Epitopic) OR TI=(difluprednate or Durezol or Epitopic) |
|  |  | *Indexes=SCI-EXPANDED, SSCI, A&HCI, CPCI-S, CPCI-SSH, BKCI-S, BKCI-SSH, ESCI, CCR-EXPANDED, IC Timespan=All years* |
| # 9 | [56](https://apps-webofknowledge-com.libaccess.lib.mcmaster.ca/summary.do?product=WOS&doc=1&qid=13&SID=8BznjzuJYXZp9nJ79Pv&search_mode=AdvancedSearch&update_back2search_link_param=yes) | AB=(desoximetasone or topicort or Topicorte) OR TI=(desoximetasone or topicort or Topicorte) |
|  |  | *Indexes=SCI-EXPANDED, SSCI, A&HCI, CPCI-S, CPCI-SSH, BKCI-S, BKCI-SSH, ESCI, CCR-EXPANDED, IC Timespan=All years* |
| # 8 | [56,440](https://apps-webofknowledge-com.libaccess.lib.mcmaster.ca/summary.do?product=WOS&doc=1&qid=11&SID=8BznjzuJYXZp9nJ79Pv&search_mode=AdvancedSearch&update_back2search_link_param=yes) | AB=(dexamethasone or BB 1101 or Decadron or Hexadrol or Alin or Fortecortin or Dexameth or Dexone or Hexadecadrol or Ozurdex or Deronil or Desamethasone or Baycuten or Aacidexam or Spersadex or Dexacortal or Gammacorten or Visumetazone or Dectancyl or Adexone or Alba-Dex or Cortidexason or Decacort or Decadrol or Desmeton or Loverine or Millicorten or Orgadrone or Aknichthol Dexa or auricularum or Auxiloson or Cortisumman or Decalix or Decameth or Decasone or Dekacort or Deltafluorene or Dexa-Mamallet or Dexafluorene or Dexalocal or Dexamecortin or Dexamethasonum or Dexamonozon or DexaposOR Desinoral or Fluorodelta or Lokalison-F or Methylfluorprednisolone or Mymethasone or Amplidermis or Anemul mono or Dexa-Rhinosan or Dexa-Scheroson or Dexa-sine or Dexacortin or Dexafarma or Dinormon or Baycadron or Maxidex or Aeroseb-Dex or Dextenza or Dexasone or Dexpak or Deca or Decaspray or Dexycu or Oradexon or Dexametasona or Corson or Dezone or Soludecadron or Solurex or disaimisong or voren or Cebedex or Dalalone or Decaject or Dekasol or Dexacen or Trabit) OR TI=(dexamethasone or BB 1101 or Decadron or Hexadrol or Alin or Fortecortin or Dexameth or Dexone or Hexadecadrol or Ozurdex or Deronil or Desamethasone or Baycuten or Aacidexam or Spersadex or Dexacortal or Gammacorten or Visumetazone or Dectancyl or Adexone or Alba-Dex or Cortidexason or Decacort or Decadrol or Desmeton or Loverine or Millicorten or Orgadrone or Aknichthol Dexa or auricularum or Auxiloson or Cortisumman or Decalix or Decameth or Decasone or Dekacort or Deltafluorene or Dexa-Mamallet or Dexafluorene or Dexalocal or Dexamecortin or Dexamethasonum or Dexamonozon or DexaposOR Desinoral or Fluorodelta or Lokalison-F or Methylfluorprednisolone or Mymethasone or Amplidermis or Anemul mono or Dexa-Rhinosan or Dexa-Scheroson or Dexa-sine or Dexacortin or Dexafarma or Dinormon or Baycadron or Maxidex or Aeroseb-Dex or Dextenza or Dexasone or Dexpak or Deca or Decaspray or Dexycu or Oradexon or Dexametasona or Corson or Dezone or Soludecadron or Solurex or disaimisong or voren or Cebedex or Dalalone or Decaject or Dekasol or Dexacen or Trabit) |
|  |  | *Indexes=SCI-EXPANDED, SSCI, A&HCI, CPCI-S, CPCI-SSH, BKCI-S, BKCI-SSH, ESCI, CCR-EXPANDED, IC Timespan=All years* |
| # 7 | [1,073](https://apps-webofknowledge-com.libaccess.lib.mcmaster.ca/summary.do?product=WOS&doc=1&qid=9&SID=8BznjzuJYXZp9nJ79Pv&search_mode=AdvancedSearch&update_back2search_link_param=yes) | AB=(clobetason or Clobetasolum or Clobetasol) OR TI=(clobetason or Clobetasolum or Clobetasol) |
|  |  | *Indexes=SCI-EXPANDED, SSCI, A&HCI, CPCI-S, CPCI-SSH, BKCI-S, BKCI-SSH, ESCI, CCR-EXPANDED, IC Timespan=All years* |
| # 6 | [73,759](https://apps-webofknowledge-com.libaccess.lib.mcmaster.ca/summary.do?product=WOS&doc=1&qid=7&SID=8BznjzuJYXZp9nJ79Pv&search_mode=AdvancedSearch&update_back2search_link_param=yes) | AB=(progesterone or prometrium or Utrogestan or crinone or Endometrin or Progesteron or cyclogest or prochieve or progest or Progesterona or Proluton or Corpus Luteum Hormones or BHR-100 or Pregnenediones) OR TI=(progesterone or prometrium or Utrogestan or crinone or Endometrin or Progesteron or cyclogest or prochieve or progest or Progesterona or Proluton or Corpus Luteum Hormones or BHR-100 or Pregnenediones) |
|  |  | *Indexes=SCI-EXPANDED, SSCI, A&HCI, CPCI-S, CPCI-SSH, BKCI-S, BKCI-SSH, ESCI, CCR-EXPANDED, IC Timespan=All years* |
| # 5 | [273,963](https://apps-webofknowledge-com.libaccess.lib.mcmaster.ca/summary.do?product=WOS&doc=1&qid=6&SID=8BznjzuJYXZp9nJ79Pv&search_mode=AdvancedSearch&update_back2search_link_param=yes) | AB=(corticosterone or Compound B) OR TI=(corticosterone or Compound B) |
|  |  | *Indexes=SCI-EXPANDED, SSCI, A&HCI, CPCI-S, CPCI-SSH, BKCI-S, BKCI-SSH, ESCI, CCR-EXPANDED, IC Timespan=All years* |
| # 4 | [3,641](https://apps-webofknowledge-com.libaccess.lib.mcmaster.ca/summary.do?product=WOS&doc=1&qid=5&SID=8BznjzuJYXZp9nJ79Pv&search_mode=AdvancedSearch&update_back2search_link_param=yes) | AB=(pregnenolone or Arthenolone or Bina-Skin or Enelone or Natolone or Pregneninolone or Pregnenolona or Pregnenolonum or Pregnetan or Pregneton or Pregnolon or Prenolon or Regnosone or Skinostelon) OR TI=(pregnenolone or Arthenolone or Bina-Skin or Enelone or Natolone or Pregneninolone or Pregnenolona or Pregnenolonum or Pregnetan or Pregneton or Pregnolon or Prenolon or Regnosone or Skinostelon) |
|  |  | *Indexes=SCI-EXPANDED, SSCI, A&HCI, CPCI-S, CPCI-SSH, BKCI-S, BKCI-SSH, ESCI, CCR-EXPANDED, IC Timespan=All years* |
| # 3 | [184,200](https://apps-webofknowledge-com.libaccess.lib.mcmaster.ca/summary.do?product=WOS&doc=1&qid=4&SID=8BznjzuJYXZp9nJ79Pv&search_mode=AdvancedSearch&update_back2search_link_param=yes) | AB=(cortisone or Adrenalex or Andreson or Compound E or Cortisal or Cortisate or Cortisona or Cortisone or Cortisonum or Cortistal or Cortivite or Cortogen or Cortone or Reichstein Fa or compound F) OR TI=(cortisone or Adrenalex or Andreson or Compound E or Cortisal or Cortisate or Cortisona or Cortisone or Cortisonum or Cortistal or Cortivite or Cortogen or Cortone or Reichstein Fa or compound F). |
|  |  | *Indexes=SCI-EXPANDED, SSCI, A&HCI, CPCI-S, CPCI-SSH, BKCI-S, BKCI-SSH, ESCI, CCR-EXPANDED, IC Timespan=All years* |
| # 2 | [130,033](https://apps-webofknowledge-com.libaccess.lib.mcmaster.ca/summary.do?product=WOS&doc=1&qid=2&SID=8BznjzuJYXZp9nJ79Pv&search_mode=AdvancedSearch&update_back2search_link_param=yes) | AB=(hydrocortisone or Acticort or Aeroseb-HC or AI3-25006 or Ala-Cort or Ala-Scalp or Alacort or Algicirtis or Amberin or Anflam or Anti-inflammatory hormone or Anucort or Anucort-HC or Anusol HC or Aquacort or Aquanil HC or Balneol-HC or Barseb HC or Basan-Corti or Beta-HC or CCRIS 5854 or Cetacort or Clear aid or Cleiton or Cobadex or Colocort or Compound F or Cort-Dome or Cortanal or Cortef or Cortenema or Cortesal or Corticreme or Cortifan or Cortiment or Cortisol or Cortisol alcohol or Cortisolonum or Cortisporin Otico or Cortispray or Cortolotion or Cortonema or Cortoxide or Cortril or Cremesone or Cremicort-H or Cutisol or Delacort or Derm-Aid or Dermacort or Dermaspray or Dermil or Dermocortal or Dermolate or Dihydrocostisone or Dioderm or Dome-cort or Domolene-HC or Efcorbin or Efcortelan or EINECS or Eldecort or Eldercort or Epicort or Epiderm or Esiderm or Evacort or Ficortril or Flexicort or Foille Insetti or Genacort or Glycort or Gyno-Cortisone or H-Cort or Heb Cort or Heb-Cort or HI-Cor or Hidalone or Hidro-colisona or Hidrocortisona or Hidrocortisona or HSDB 3339 or Hycort or Hycortol or Hycortole or Hydrasson or Hydro-Adreson or Hydro-Colisona or Hydro-RX or Hydrocorticosterone or Hydrocortisonum or Hydrocortisyl or Hydrocortone or Hydroxycortisone or Hytisone or Hytone or Idrocortisone or Incortin-H or Incortin-hydrogen or Kendall's compound F or Komed HC or Kyypakkaus or Lacticare-HC or Lactisona or Lubricort or Maintasone or Meusicort or Mildison or Milliderm or Neosporin-H Ear or Nogenic HC or NSC 10483 or Nutracort or Optef or Otosone-F or Penecort or Permicort or Polcort H or Preparation H Hydrocortisone or Prevex HC or Proctocort or Proctofoam or Proctosol-HC or Proctozone HC or Rectasol-HC or Rectoid or Reichstein's substance M or Remederm HC or Sanatison or Scalp-Cort or Scalpicin Capilar or Schericur or Scheroson F or Sigmacort or Signef or Stie-Cort or Stiefcorcil or Synacort or Systral Hydrocort or Tarcortin or Texacort or Timocort or Transderma H or Traumaide or Uniderm) OR TI=(hydrocortisone or Acticort or Aeroseb-HC or AI3-25006 or Ala-Cort or Ala-Scalp or Alacort or Algicirtis or Amberin or Anflam or Anti-inflammatory hormone or Anucort or Anucort-HC or Anusol HC or Aquacort or Aquanil HC or Balneol-HC or Barseb HC or Basan-Corti or Beta-HC or CCRIS 5854 or Cetacort or Clear aid or Cleiton or Cobadex or Colocort or Compound F or Cort-Dome or Cortanal or Cortef or Cortenema or Cortesal or Corticreme or Cortifan or Cortiment or Cortisol or Cortisol alcohol or Cortisolonum or Cortisporin Otico or Cortispray or Cortolotion or Cortonema or Cortoxide or Cortril or Cremesone or Cremicort-H or Cutisol or Delacort or Derm-Aid or Dermacort or Dermaspray or Dermil or Dermocortal or Dermolate or Dihydrocostisone or Dioderm or Dome-cort or Domolene-HC or Efcorbin or Efcortelan or EINECS or Eldecort or Eldercort or Epicort or Epiderm or Esiderm or Evacort or Ficortril or Flexicort or Foille Insetti or Genacort or Glycort or Gyno-Cortisone or H-Cort or Heb Cort or Heb-Cort or HI-Cor or Hidalone or Hidro-colisona or Hidrocortisona or Hidrocortisona or HSDB 3339 or Hycort or Hycortol or Hycortole or Hydrasson or Hydro-Adreson or Hydro-Colisona or Hydro-RX or Hydrocorticosterone or Hydrocortisonum or Hydrocortisyl or Hydrocortone or Hydroxycortisone or Hytisone or Hytone or Idrocortisone or Incortin-H or Incortin-hydrogen or Kendall's compound F or Komed HC or Kyypakkaus or Lacticare-HC or Lactisona or Lubricort or Maintasone or Meusicort or Mildison or Milliderm or Neosporin-H Ear or Nogenic HC or NSC 10483 or Nutracort or Optef or Otosone-F or Penecort or Permicort or Polcort H or Preparation H Hydrocortisone or Prevex HC or Proctocort or Proctofoam or Proctosol-HC or Proctozone HC or Rectasol-HC or Rectoid or Reichstein's substance M or Remederm HC or Sanatison or Scalp-Cort or Scalpicin Capilar or Schericur or Scheroson F or Sigmacort or Signef or Stie-Cort or Stiefcorcil or Synacort or Systral Hydrocort or Tarcortin or Texacort or Timocort or Transderma H or Traumaide or Uniderm) |
|  |  | *Indexes=SCI-EXPANDED, SSCI, A&HCI, CPCI-S, CPCI-SSH, BKCI-S, BKCI-SSH, ESCI, CCR-EXPANDED, IC Timespan=All years* |
| # 1 | [19,853](https://apps-webofknowledge-com.libaccess.lib.mcmaster.ca/summary.do?product=WOS&doc=1&qid=1&SID=8BznjzuJYXZp9nJ79Pv&search_mode=AdvancedSearch&update_back2search_link_param=yes) | AB=(aldosterone or Aldocorten or Aldocortene or Aldocortin or Aldosterona or Aldosterone or Aldosteronum or Electrocortin or Elektrocortin or Reichstein X) |
|  |  | *Indexes=SCI-EXPANDED, SSCI, A&HCI, CPCI-S, CPCI-SSH, BKCI-S, BKCI-SSH, ESCI, CCR-EXPANDED, IC Timespan=All years* |

1304 citations

**Appendix 6. WHO International Clinical Trials Registry Platform (ICTRP) Search Strategy**

Basic search (advanced searching was not functioning)

One of the following: “heart arrest” OR heart standstill OR sudden cardiac death OR sudden death OR cardiopulmonary resuscitation OR shock reversal OR cardiogenic shock

AND

One of the following: corticosteroid OR steroid

(without synonyms box checked)

Phases: “all”

68 studies

**Appendix 7. Risk of Bias Table**

Table assessing the risk of bias for included randomized control trials comparing corticosteroids to placebo for patients post cardiac arrest.

| Study (author, year) | Randomization | Deviations | Missing Outcomes | Outcome Measurement | Selective Reporting | Overall ROB |
| --- | --- | --- | --- | --- | --- | --- |
| Mentzelopoulos, 2013 | Low | Low | Low | Low | Low | Low |
| Bolvadri, 2016 | Low | Probably High | Probably Low | Probably High | Probably Low | High |
| Donnino, 2016 | Probably Low | Probably Low | Low | Probably Low | Probably High | High |
| Mentzelopoulos, 2009 | Low | Low | Low | Low | Low | Low |
| Paris, 1984 | Probably Low | Probably High | Probably Low | Probably High | High | High |
| Metz, 1986 | Probably Low | Probably High | Probably High | Probably High | High | High |
| Andersen, 2021 | Low | Probably High | Low | Low | Low | Low |
| Rafiei, 2022 | Probably Low | Probably Low | Low | Probably High | Low | Low |

**Appendix 8. Forest Plot for All Outcomes**

**Supplement Figure 1. Forest plot. Mortality closest to 28 days:**

Comparing corticosteroids and placebo for the outcome of mortality closest to 28 days; results are shown by using the random-effects model with relative risk and 95% confidence intervals (CI)


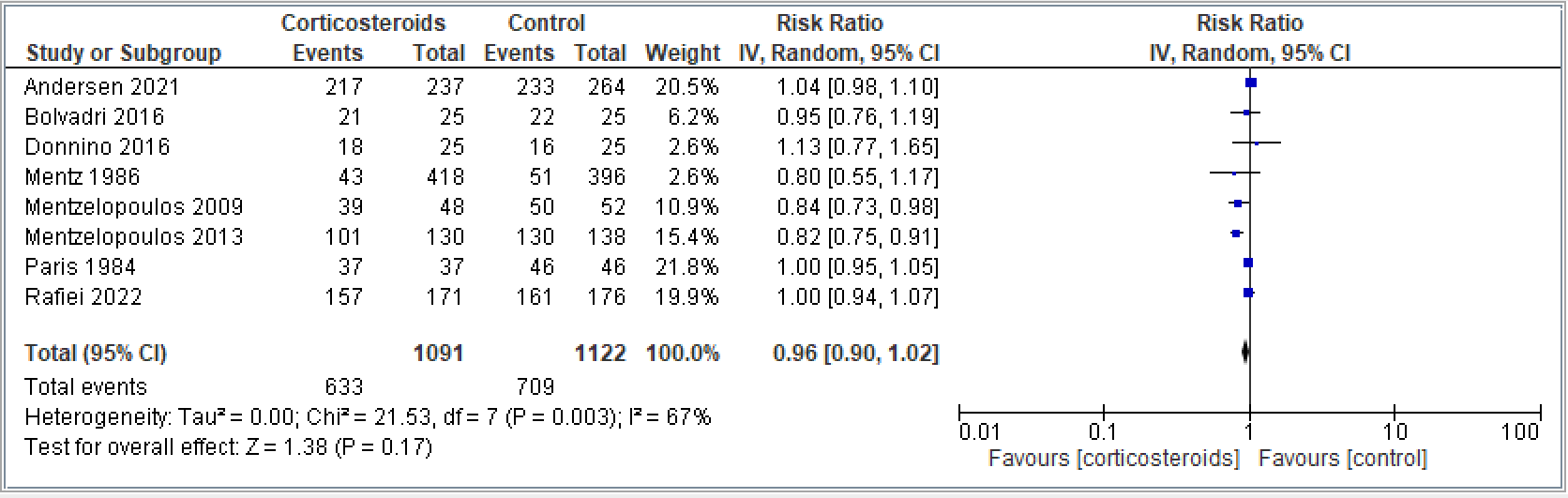


**Supplement Figure 2. Forest plot. Return of Spontaneous Circulation:**

Comparing corticosteroids and placebo for the outcome of return of spontaneous circulation; results are shown by using the random-effects model with relative risk and 95% confidence intervals (CI)


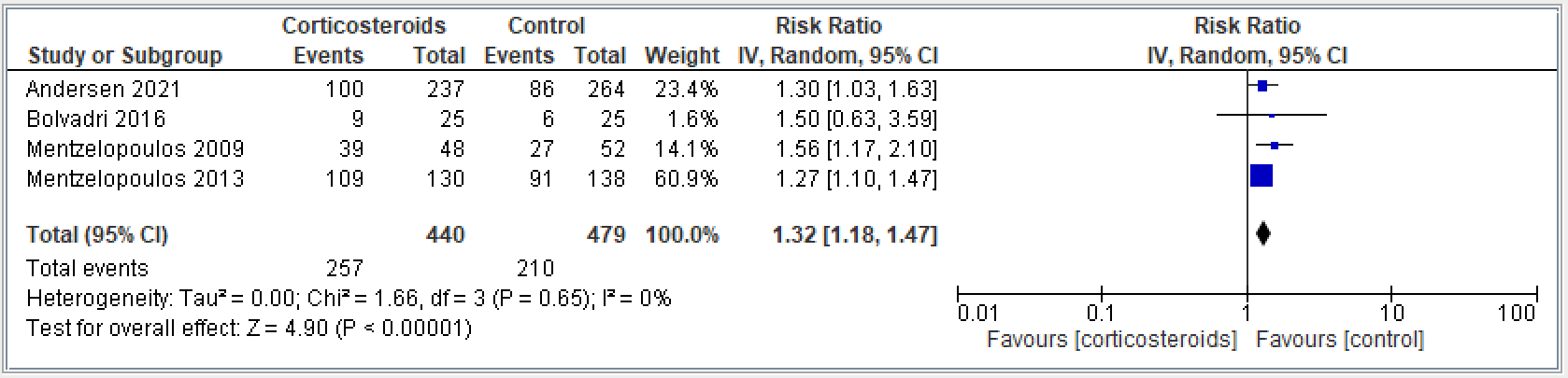


**Supplement Figure 3. Forest plot. Survival with Good Functional Outcome:**

Comparing corticosteroids and placebo for the outcome of survival with good functional outcome; results are shown by using the random-effects model with relative risk and 95% confidence intervals (CI)


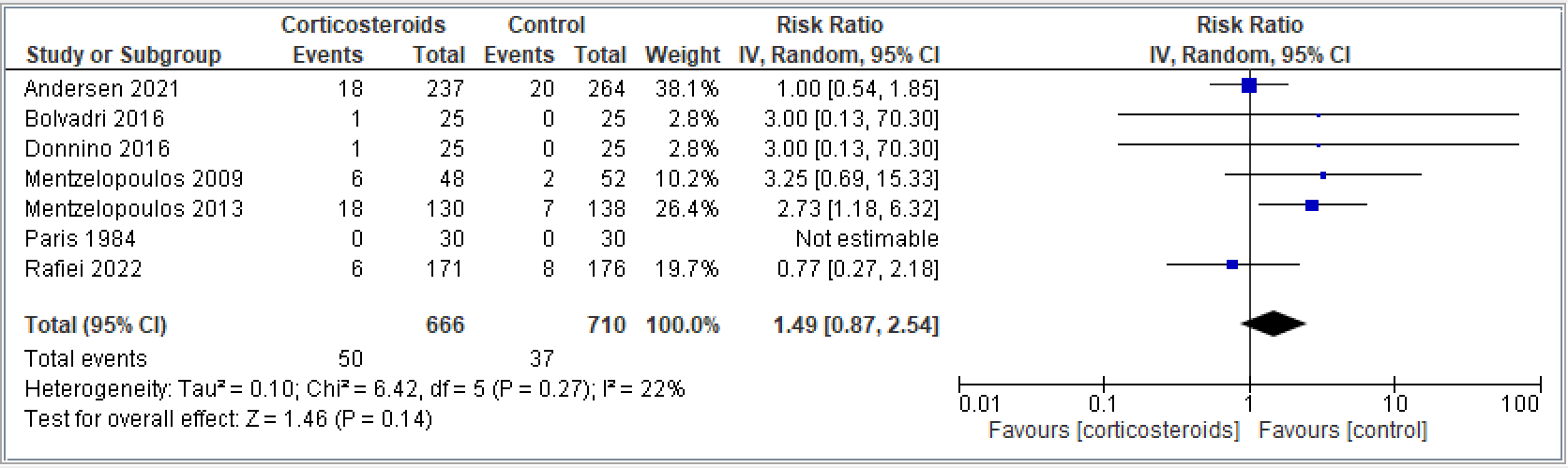


**Supplement Figure 4. Forest plot. Renal Failure:**

Comparing corticosteroids and placebo for the outcome of renal failure; results are shown by using the random-effects model with relative risk and 95% confidence intervals (CI)


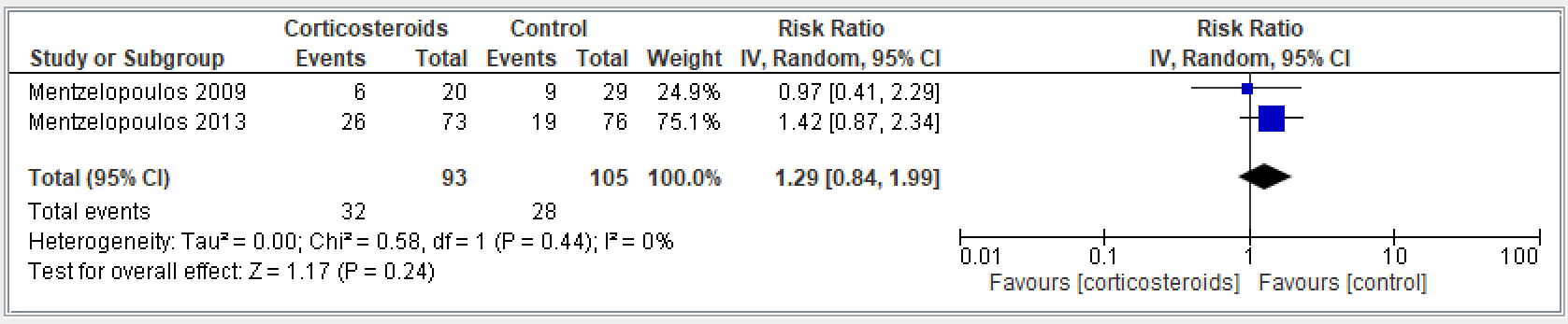


**Supplement Figure 5. Forest plot. Peritonitis:**

Comparing corticosteroids and placebo for the outcome of renal failure; results are shown by using the random-effects model with relative risk and 95% confidence intervals (CI)


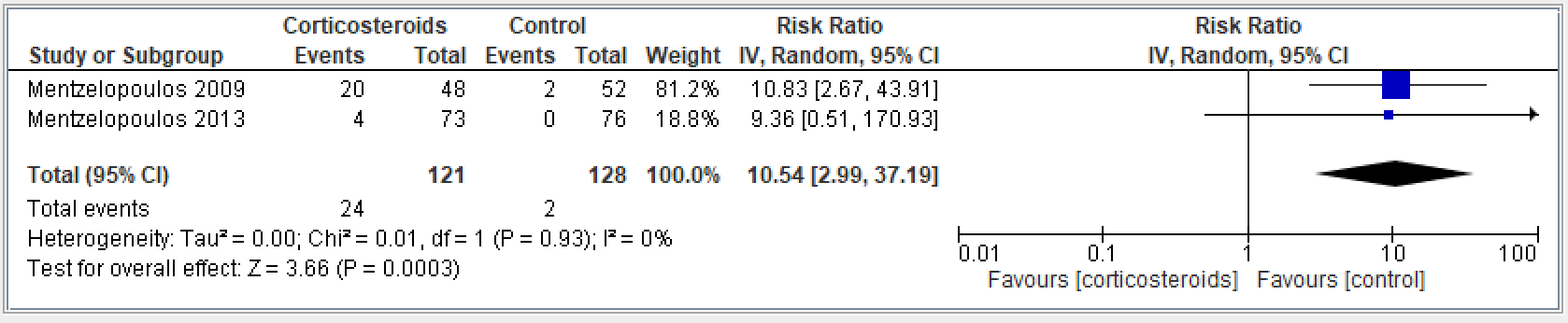


**Supplement Figure 6. Forest plot. Bleeding:**

Comparing corticosteroids and placebo for the outcome of bleeding; results are shown by using the random-effects model with relative risk and 95% confidence intervals (CI)


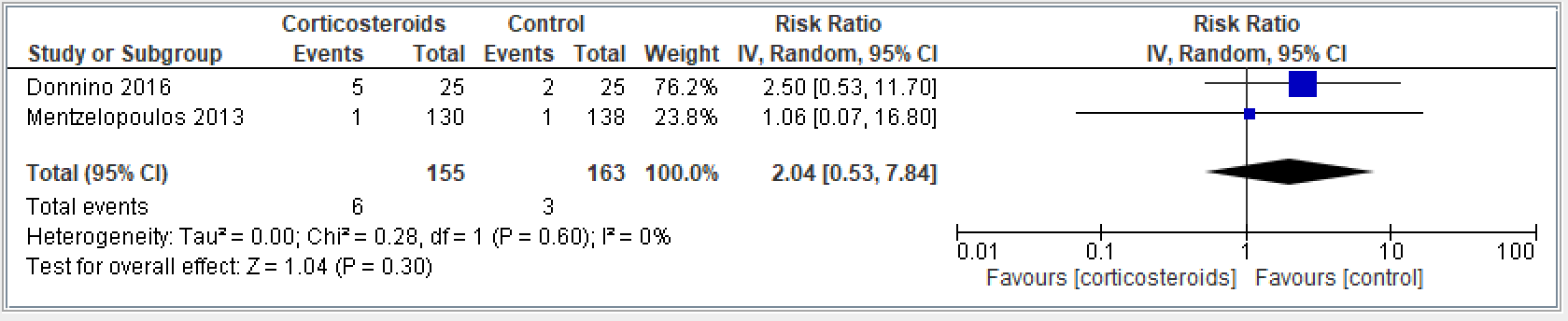


**Supplement Figure 7. Forest plot. Ventilator Assisted Pneumonia:**

Comparing corticosteroids and placebo for the outcome of ventilator assisted pneumonia; results are shown by using the random-effects model with relative risk and 95% confidence intervals (CI)


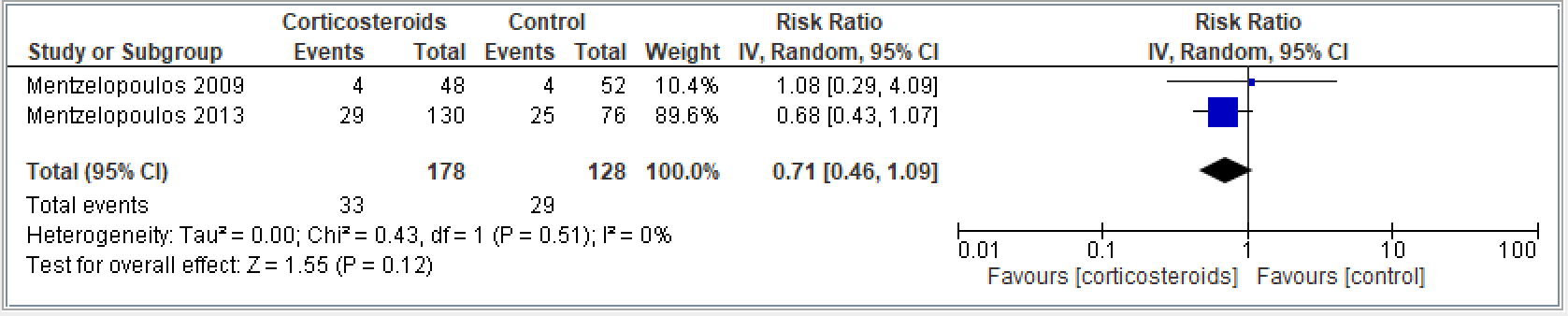


**Appendix 9. Subgroup Analysis Forest Plots**

**
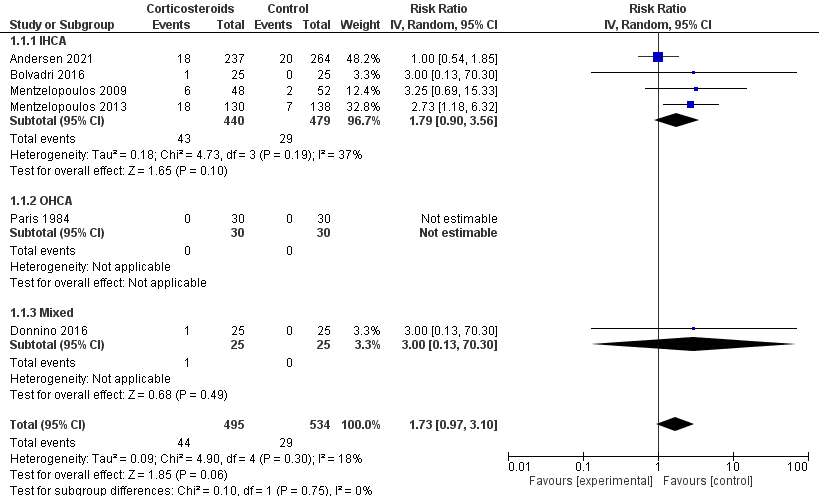
Supplement Figure 1. Forest plot. Mortality:**

**Supplement Figure 2. Forest plot. Survival with Good Functional Outcome:**

**
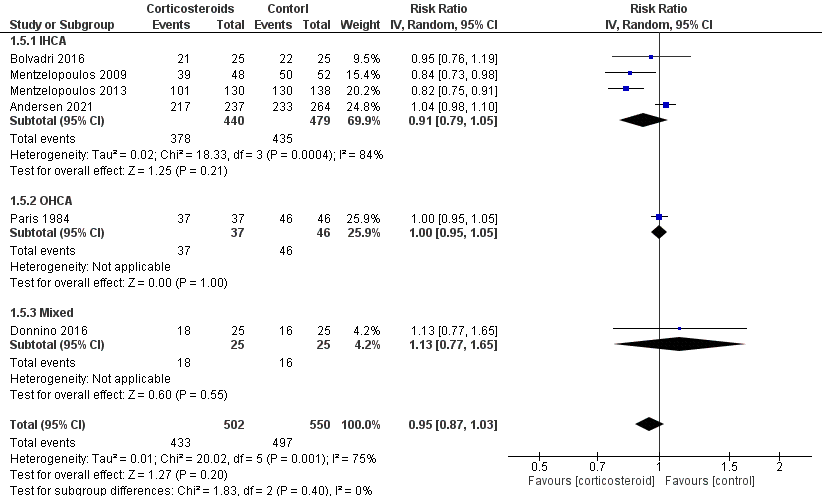
**

**Appendix 10. Trail Sequential Analysis (TSA)**


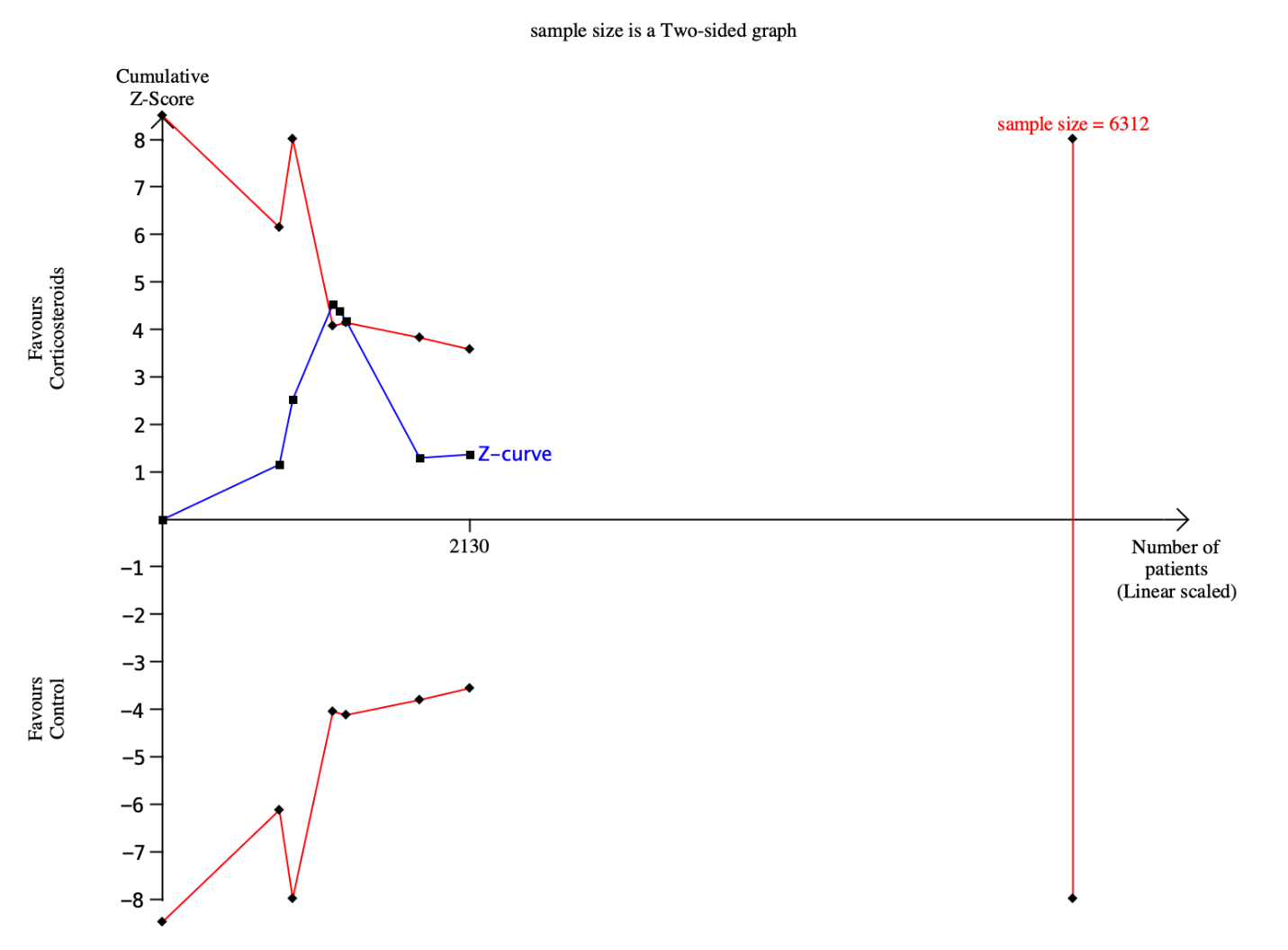


**Appendix 11. PRISMA 2020 Table**

| **Section and Topic** | **Item #** | **Checklist item** | **Location where item is reported** |
| --- | --- | --- | --- |
| **TITLE** | | |  |
| Title | 1 | Identify the report as a systematic review. | Page 1 |
| **ABSTRACT** | | |  |
| Abstract | 2 | See the PRISMA 2020 for Abstracts checklist. | Page 2-3 |
| **INTRODUCTION** | | |  |
| Rationale | 3 | Describe the rationale for the review in the context of existing knowledge. | Page 3 |
| Objectives | 4 | Provide an explicit statement of the objective(s) or question(s) the review addresses. | Page 3-4 |
| **METHODS** | | |  |
| Eligibility criteria | 5 | Specify the inclusion and exclusion criteria for the review and how studies were grouped for the syntheses. | Page 4-5 |
| Information sources | 6 | Specify all databases, registers, websites, organisations, reference lists and other sources searched or consulted to identify studies. Specify the date when each source was last searched or consulted. | Page 4 |
| Search strategy | 7 | Present the full search strategies for all databases, registers and websites, including any filters and limits used. | Page 4; Supplementary (1-4) |
| Selection process | 8 | Specify the methods used to decide whether a study met the inclusion criteria of the review, including how many reviewers screened each record and each report retrieved, whether they worked independently, and if applicable, details of automation tools used in the process. | Page 4-5 |
| Data collection process | 9 | Specify the methods used to collect data from reports, including how many reviewers collected data from each report, whether they worked independently, any processes for obtaining or confirming data from study investigators, and if applicable, details of automation tools used in the process. | Page 4 |
| Data items | 10a | List and define all outcomes for which data were sought. Specify whether all results that were compatible with each outcome domain in each study were sought (e.g. for all measures, time points, analyses), and if not, the methods used to decide which results to collect. | Page 5 |
|  | 10b | List and define all other variables for which data were sought (e.g. participant and intervention characteristics, funding sources). Describe any assumptions made about any missing or unclear information. | Page 4-5 |
| Study risk of bias assessment | 11 | Specify the methods used to assess risk of bias in the included studies, including details of the tool(s) used, how many reviewers assessed each study and whether they worked independently, and if applicable, details of automation tools used in the process. | Page 5 |
| Effect measures | 12 | Specify for each outcome the effect measure(s) (e.g. risk ratio, mean difference) used in the synthesis or presentation of results. | **Not Applicable** |
| Synthesis methods | 13a | Describe the processes used to decide which studies were eligible for each synthesis (e.g. tabulating the study intervention characteristics and comparing against the planned groups for each synthesis (item #5)). | Page 5 |
|  | 13b | Describe any methods required to prepare the data for presentation or synthesis, such as handling of missing summary statistics, or data conversions. | **Not Applicable** |
|  | 13c | Describe any methods used to tabulate or visually display results of individual studies and syntheses. | **Not Applicable** |
|  | 13d | Describe any methods used to synthesize results and provide a rationale for the choice(s). If meta-analysis was performed, describe the model(s), method(s) to identify the presence and extent of statistical heterogeneity, and software package(s) used. | Page 5 |
|  | 13e | Describe any methods used to explore possible causes of heterogeneity among study results (e.g. subgroup analysis, meta-regression). | Page 5 |
|  | 13f | Describe any sensitivity analyses conducted to assess robustness of the synthesized results. | **Not Applicable** |
| Reporting bias assessment | 14 | Describe any methods used to assess risk of bias due to missing results in a synthesis (arising from reporting biases). | **Not Applicable** |
| Certainty assessment | 15 | Describe any methods used to assess certainty (or confidence) in the body of evidence for an outcome. | Page 5 |
| **RESULTS** | | |  |
| Study selection | 16a | Describe the results of the search and selection process, from the number of records identified in the search to the number of studies included in the review, ideally using a flow diagram. | Page 6 |
|  | 16b | Cite studies that might appear to meet the inclusion criteria, but which were excluded, and explain why they were excluded. | Not Applicable |
| Study characteristics | 17 | Cite each included study and present its characteristics. | Page 6 |
| Risk of bias in studies | 18 | Present assessments of risk of bias for each included study. | Supplementary (7) |
| Results of individual studies | 19 | For all outcomes, present, for each study: (a) summary statistics for each group (where appropriate) and (b) an effect estimate and its precision (e.g. confidence/credible interval), ideally using structured tables or plots. | Supplementary (8) |
| Results of syntheses | 20a | For each synthesis, briefly summarise the characteristics and risk of bias among contributing studies. | Page 7 |
|  | 20b | Present results of all statistical syntheses conducted. If meta-analysis was done, present for each the summary estimate and its precision (e.g. confidence/credible interval) and measures of statistical heterogeneity. If comparing groups, describe the direction of the effect. | Page 7-8 |
|  | 20c | Present results of all investigations of possible causes of heterogeneity among study results. | Page 7-8 |
|  | 20d | Present results of all sensitivity analyses conducted to assess the robustness of the synthesized results. | **Not Applicable** |
| Reporting biases | 21 | Present assessments of risk of bias due to missing results (arising from reporting biases) for each synthesis assessed. | Supplementary (7) |
| Certainty of evidence | 22 | Present assessments of certainty (or confidence) in the body of evidence for each outcome assessed. | Table 2 |
| **DISCUSSION** | | |  |
| Discussion | 23a | Provide a general interpretation of the results in the context of other evidence. | Page 8-9 |
|  | 23b | Discuss any limitations of the evidence included in the review. | Page 10-11 |
|  | 23c | Discuss any limitations of the review processes used. | Page 10-11 |
|  | 23d | Discuss implications of the results for practice, policy, and future research. | Page 10 |
| **OTHER INFORMATION** | | |  |
| Registration and protocol | 24a | Provide registration information for the review, including register name and registration number, or state that the review was not registered. | Page 4 |
|  | 24b | Indicate where the review protocol can be accessed, or state that a protocol was not prepared. | Page 4 |
|  | 24c | Describe and explain any amendments to information provided at registration or in the protocol. | **Not Applicable** |
| Support | 25 | Describe sources of financial or non-financial support for the review, and the role of the funders or sponsors in the review. | **Title Page** |
| Competing interests | 26 | Declare any competing interests of review authors. | **Conflict of Interest Document** |
| Availability of data, code and other materials | 27 | Report which of the following are publicly available and where they can be found: template data collection forms; data extracted from included studies; data used for all analyses; analytic code; any other materials used in the review. | **Not Applicable** |

*From:*  Page MJ, McKenzie JE, Bossuyt PM, Boutron I, Hoffmann TC, Mulrow CD, et al. The PRISMA 2020 statement: an updated guideline for reporting systematic reviews. BMJ 2021;372:n71. doi: 10.1136/bmj.n71

For more information, visit: <http://www.prisma-statement.org/>
